# Impact of tight blood glucose control within normal fasting ranges with insulin titration prescribed by the Leuven algorithm in adult critically ill patients: the TGC-fast randomized controlled trial

**DOI:** 10.1186/s13063-022-06709-8

**Published:** 2022-09-19

**Authors:** Jan Gunst, Liese Mebis, Pieter J. Wouters, Greet Hermans, Jasperina Dubois, Alexander Wilmer, Eric Hoste, Dominique Benoit, Greet Van den Berghe

**Affiliations:** 1 Laboratory of Intensive Care Medicine, Department of Cellular and Molecular Medicine, KU Leuven, Leuven, Belgium; 2 Department of Intensive Care Medicine, University Hospitals Leuven, Leuven, Belgium; 3 Medical Intensive Care unit, Clinical Department of General Internal Medicine, University Hospitals Leuven, Leuven, Belgium; 4 Department of Anesthesiology and Intensive Care Medicine, Jessa Hospitals, Hasselt, Belgium; 5 Department of Intensive Care Medicine, University Hospitals Ghent, Ghent, Belgium

**Keywords:** Hyperglycemia, Insulin, Glucose control, Critical illness, Intensive care, Parenteral nutrition

## Abstract

**Background:**

It remains controversial whether critical illness-related hyperglycemia should be treated or not, since randomized controlled trials (RCTs) have shown context-dependent outcome effects. Whereas pioneer RCTs found improved outcome by normalizing blood glucose in patients receiving early parenteral nutrition (PN), a multicenter RCT revealed increased mortality in patients not receiving early PN. Although withholding early PN has become the feeding standard, the multicenter RCT showing harm by tight glucose control in this context has been criticized for its potentially unreliable glucose control protocol. We hypothesize that tight glucose control is effective and safe using a validated protocol in adult critically ill patients not receiving early PN.

**Methods:**

The TGC-fast study is an investigator-initiated, multicenter RCT. Patients unable to eat, with need for arterial and central venous line and without therapy restriction, are randomized upon ICU admission to tight (80–110 mg/dl) or liberal glucose control (only initiating insulin when hyperglycemia >215 mg/dl, and then targeting 180–215 mg/dl). Glucose measurements are performed on arterial blood by a blood gas analyzer, and if needed, insulin is only administered continuously through a central venous line. If the arterial line is no longer needed, glucose is measured on capillary blood. In the intervention group, tight control is guided by the validated LOGIC-Insulin software. In the control arm, a software alert is used to maximize protocol compliance. The intervention is continued until ICU discharge, until the patient is able to eat or no longer in need of a central venous line, whatever comes first. The study is powered to detect, with at least 80% power and a 5% alpha error rate, a 1-day difference in ICU dependency (primary endpoint), and a 1.5% increase in hospital mortality (safety endpoint), for which 9230 patients need to be included. Secondary endpoints include acute and long-term morbidity and mortality, and healthcare costs. Biological samples are collected to study potential mechanisms of organ protection.

**Discussion:**

The ideal glucose target for critically ill patients remains debated. The trial will inform physicians on the optimal glucose control strategy in adult critically ill patients not receiving early PN.

**Trial registration:**

ClinicalTrials.gov NCT03665207. Registered on 11 September 2018.

**Supplementary Information:**

The online version contains supplementary material available at 10.1186/s13063-022-06709-8.

## Administrative information

Note: the numbers in curly brackets in this protocol refer to SPIRIT checklist item numbers. The order of the items has been modified to group similar items (see http://www.equator-network.org/reporting-guidelines/spirit-2013-statement-defining-standard-protocol-items-for-clinical-trials/).

**Table:** 

Title {1}	Impact of tight blood glucose control within normal fasting ranges with insulin titration prescribed by the Leuven algorithm in adult critically ill patients: the TGC-fast randomized controlled trial
Trial registration {2a and 2b}.	EudraCT: 2018-000756-17 clinicaltrials.gov: NCT03665207, registered September 11, 2018; https://www.clinicaltrials.gov/ct2/show/NCT03665207
Protocol version {3}	Version 1.5, 12-01-2021 – approved 9-03-2021 Revision chronology: Version 1.2, 18-05-2018 – approved 30-05-2018; original protocol Version 1.4, 15-05-2019 – approved 26-06-2019; amendment 1 Summary of major changes: - Extension of the time window of obtaining deferred informed consent - Specification of certain exclusion criteria. Patients refusing blood transfusion upon intensive care unit (ICU) admission are considered as having a therapy restriction. Patients in need of high doses of parenteral glucose upon ICU admission are considered as being in need for early parenteral nutrition. For the same reason, patients planned to receive peritoneal dialysis are not included. Specification that inclusion in an investigational medicinal product-randomized controlled trial (RCT) of which the principal investigator indicates that co-inclusion is prohibited, is an exclusion criterion. Version 1.5, 12-01-2021 – approved 9-03-2021; amendment 2 Summary of major changes: - Addition of atrial fibrillation and major adverse cardiovascular events as secondary endpoints, and of baseline cardiovascular risk factors and comorbidities (in selected centers) - Addition that, in selected centers, pleural and pericardial fluid samples will be collected in patients admitted after cardiac surgery - Change of the time point of electrophysiological and morphological assessment of muscle wasting and weakness, and of muscle biopsy: day 7-14-21 instead of day 14-21-28; waiver of informed consent for ultrasound evaluation of muscle
Funding {4}	TBM project (Applied Biomedical Research with a Primary Social finality) grant by Research Foundation – Flanders (T003617N) to GVdB, JG and DB Structural research financing by the Methusalem programme of the Flemish Government through the University of Leuven (METH14/06) to GVdB European Research Council (ERC) Advanced Grant from the Horizon 2020 Program of the EU to GVdB (AdvG-2017-785809) European Research Council (ERC) Advanced Grant of GVdB Postdoctoral research fellowship to JG, granted by the Clinical Research and Education Council of the University Hospitals Leuven Fundamental Clinical Research fellowship to GH, granted by the Research Foundation – Flanders (1805121N).
Author details {5a}	^1^ Laboratory of Intensive Care Medicine, Department of Cellular and Molecular Medicine, KU Leuven, Leuven, Belgium ^2^ Department of Intensive Care Medicine, University Hospitals Leuven, Leuven, Belgium ^3^ Medical Intensive Care unit, Clinical Department of General Internal Medicine, University Hospitals Leuven, Leuven, Belgium ^4^ Department of Anesthesiology and Intensive Care Medicine, Jessa Hospitals, Hasselt, Belgium ^5^ Department of Intensive Care Medicine, University Hospitals Ghent, Ghent, Belgium
Name and contact information for the trial sponsor {5b}	KU Leuven Contact person: Greet Van den Berghe MD, PhD, Herestraat 49, 3000 Leuven, Belgium. Phone +32 16 344021. Email greet.vandenberghe@kuleuven.be
Role of sponsor {5c}	The study is an investigator-initiated randomized controlled trial (academic sponsor). The funders of the study have no role in study design, data collection, data management, data analysis, writing of the report, or the decision to submit the report for publication.

## Introduction

### Background and rationale {6a}

Acute medical illnesses, major trauma, and extensive surgery induce profound physiological alterations that may require admission to an intensive care unit (ICU) to prevent or treat life-threatening manifestations and complications hereof, in order to restore or maintain homeostasis. Thanks to advances in intensive care medicine, critically ill patients now usually survive conditions that were previously lethal. Still, a considerable number of patients do not recover swiftly and remain dependent on intensive care for weeks to months. The longer the ICU stay, the higher the risk of death, and also ICU survivors have an important long-term legacy [1]. The costs for society of intensive care are enormous and predominantly driven by the duration of ICU stay and by infectious complications [2].

Hyperglycemia is present in virtually every adult patient in the ICU, and the degree of hyperglycemia is related to the risk of adverse outcome [3, 4]. However, whether this relationship is causal or merely reflective of more severe insulin resistance in sicker patients remains debated, since randomized controlled trials (RCTs) have yielded at first sight contradictory results [5–12]. Indeed, in 3 landmark single center RCTs performed in Leuven, Belgium, lowering blood glucose to the healthy fasting range (50–80 mg/dl [2.8–4.4 mmol/l] for infants, 70–100 mg/dl [3.9–5.6 mmol/l] for children older than 1 year, and 80–110 mg/dl [4.4–6.1 mmol/l] for adults) improved short- and long-term morbidity and mortality and reduced use of healthcare resources as compared to tolerating hyperglycemia up to 215 mg/dl (11.9 mmol/l) [5–10]. In particular, the duration of ICU dependency was reduced substantially. However, a subsequent large pragmatic multicenter RCT found increased mortality with TGC attributable to more hypoglycemia [11, 12]. These opposing results may be explained by methodological differences between the pioneer studies and the generalizability trial. In this regard, the multicenter RCT has been criticized for the use of an unvalidated and potentially unreliable glucose control protocol that included potentially inaccurate glucose measurements and insulin boluses [13, 14]. This likely increased the risk of hypoglycemia and of glucose variability, which is associated with poor outcome [15]. In contrast, the Leuven protocol was well standardized by ensuring accurate glucose measurements and by avoiding insulin boluses, and glucose control was performed by extensively trained nurses [16]. However, unlike in the generalizability RCT, all patients in the pioneer studies in Leuven received early parenteral nutrition when enteral feeding was insufficient to meet the caloric target [5, 8, 9]. This feeding strategy, which increases the degree of hyperglycemia, has been abandoned in more recent years after multicenter RCTs showed prolonged ICU dependency, also when feeding-induced hyperglycemia was treated [17, 18]. It currently remains unknown whether tight glucose control is effective and safe when provided with an accurate protocol that avoids large glucose fluctuations and hypoglycemia, in the absence of early parenteral feeding.

To investigate this, we are performing a multicenter RCT—the TGC-fast RCT—that is adequately powered for patient-centered and economic endpoints. Patients are randomly allocated to tight glucose control to target normal fasting glucose concentrations with insulin versus tolerating hyperglycemia up to a predefined level. Tight glucose control is guided by our previously developed and validated computerized LOGIC-Insulin algorithm that has shown to be superior to nurse-guided glucose control, with virtually prevention of hypoglycemia [19]. The efficacy and safety of the LOGIC-Insulin algorithm has been confirmed in a multicenter context by members of the TGC-fast consortium [20].

### Objectives {7}

The main objective of the TGC-fast RCT is to test the hypothesis that in adult critically ill patients receiving an evidence-based, restrictive feeding regimen (no parenteral nutrition in the first week of critical illness), targeting normal blood glucose concentrations (80–110 mg/dl, 4.4–6.1 mmol/l), reduces short-term morbidity and associated dependency on intensive care as compared with tolerating hyperglycemia up to 215 mg/dl (11.9 mmol/l). The null hypothesis is that there will be no difference in the primary outcome between the two strategies. The study is two-tailed so that it is also powered to detect a negative impact of targeting normal blood glucose concentrations. As a safety endpoint, hospital mortality will be monitored. The analyses will be done unadjusted as well as adjusted for risk factors.

Secondary objectives are to study the long-term impact of the intervention (morbidity and mortality), to study the economic impact (healthcare resources) of the intervention, and to study the underlying mechanisms that may explain an eventual benefit (or harm). This will be achieved by studying the effects of treatment allocation on metabolic, endocrine, inflammatory, coagulation, cardiac and (epi)genetic markers in body fluid, and tissue samples of critically ill patients.

### Trial design {8}

The study is a multicenter, parallel group randomized controlled study that will study the efficacy and safety of tight glucose control in the absence of early parenteral nutrition. Patients are randomized in a 1:1 ratio. Patients and family members are blinded to treatment assignment. Due to the nature of the study, blinding bedside physicians and nurses is not possible. Outcome assessors are blinded, however.

## Methods: participants, interventions, and outcomes

### Study setting {9}

The study is performed in different centers in Belgium, including both university and non-university hospitals. The study sites are listed on clinicaltrials.gov (NCT03665207).

### Eligibility criteria {10}

Inclusion criteria:

All adult (≥18 years of age) patients admitted to one of the participating intensive care units

Exclusion criteria:

- Patients with a do not resuscitate order at the time of ICU admission
- Patients expected to die within 12 h after ICU admission (moribund patients)
- Patients able to receive oral feeding (not critically ill)
- Patients without arterial and without central venous line and without imminent need to place it as part of ICU management (not critically ill)
- Patients previously included in the trial (when readmission is within 48 h post ICU discharge, the trial intervention will be resumed)
- Inclusion in an investigational medicinal product-RCT of which the principal investigator indicates that co-inclusion specifically in the TGC-fast RCT is prohibited
- Patients transferred from a non-participating ICU with a pre-admission ICU stay >7 days
- Patients planned to receive parenteral nutrition during the first week in ICU
- Patients suffering from diabetic ketoacidosis or hyperosmolar hyperglycemic state on ICU admission
- Patients with inborn metabolic diseases
- Patients with insulinoma
- Patients known to be pregnant or lactating
- Informed consent refusal

### Who will take informed consent? {26a}

Written informed consent is obtained from the patient or patient representative. Informed consent is obtained by the treating physician. Depending on whether the admission is elective or urgent, and whether one is able to contact the patient before ICU admission, informed consent is obtained prior to or shortly after ICU admission. For planned ICU admissions after elective procedures, informed consent is asked prior to the procedure when possible. For urgently admitted patients, obtaining informed consent prior to ICU admission is impossible. As a blood glucose control strategy has to be initiated upon admission, treatment allocation is done after assessment of the patient for eligibility by the attending physician within the time frame of two hours (deferred informed consent when one was not able to contact the patient or patient representative before ICU admission). In the latter case (deferred informed consent), maximal efforts are done to obtain a written informed consent form as soon as possible, and preferably within 72 h after ICU admission. A duplicate of the signed informed consent form is given to the patient or the patient representative. Especially in urgently admitted patients, informed consent is often obtained from the patient’s representative. As regaining consciousness and mental competence often occurs gradually and slowly in these patients, it is very difficult to determine at what time the patient is able to give a valid informed consent. In addition, recovery can take from several days to several months. Often, patients are only able to give a valid consent after discharge from ICU, at what time the intervention is already terminated. Therefore, we give an opting out form to the patient representative, who is asked to give it to the patient once he/she is well recovered. With this document, the patient has the possibility to terminate the study intervention (if still applicable) and/or withdraw from further data collection.

### Additional consent provisions for collection and use of participant data and biological specimens {26b}

In long-stay ICU patients (≥7 days in ICU) in selected centers, additional informed consent is obtained to perform electrophysiological tests (nerve conduction, needle electromyography and direct muscle stimulation), ultrasound quantification of muscle thickness, and/or muscle biopsy at days 7±1, 14±1, 21±1, and 28±1, as long as the patient remains in ICU.

## Interventions

### Explanation for the choice of comparators {6b}

The ideal blood glucose target remains debated, and local blood glucose control practices vary widely [21–24]. In the control group of the TGC-fast RCT (liberal blood glucose control), hyperglycemia is only treated when above 215 mg/dl (11.9 mmol/l) and hence exceeding the renal threshold, above which obvious complications may ensue [16]. This threshold to initiate insulin treatment in the control group was also used in the pioneer RCTs investigating tight versus liberal blood glucose control [5, 8, 9].

In both study groups, blood glucose concentrations are measured by an on-site blood gas analyzer in undiluted blood drawn from the arterial line. Glucose measurement on arterial blood using an on-site blood gas analyzer yields both a fast and accurate measurement of blood glucose in critically ill patients [14]. When the arterial catheter is no longer needed (for medical reasons), blood glucose is measured on capillary blood using a glucometer with a validated performance. Measurement of the blood glucose concentration on blood drawn from the (central) venous catheter through which insulin and glucose are administered is not allowed due to potential interference with the measurement. In both study groups, insulin is only infused through continuous intravenous infusion through a central venous catheter by a syringe pump, usually in concentrations of 50 IU in 50 mL 0.9% NaCl. The concentration can be increased to 100 IU in 50 mL NaCl 0.9% in case of a high insulin need. No boluses of insulin are allowed.

In the liberal glucose control group (control group), insulin is initiated when blood glucose concentrations exceed 215 mg/dl (11.9 mmol/l) on two consecutive measurements, with the dose adjusted by the nurses/physicians to maintain concentrations between 180 and 215 mg/dl (10–11.9 mmol/l). When blood glucose drops below 180 mg/dl (10 mmol/l), insulin infusion is stopped (except for type 1 diabetics). In type 1 diabetics, insulin is initiated after the first blood glucose measurement above 215 mg/dl (11.9 mmol/l) and the infusion rate is adjusted to maintain blood glucose concentrations between 180 and 215 mg/dl (between 10 and 11.9 mmol/l). Blood glucose is measured minimum 4 times per day. The management of eventual hypoglycemia is at the discretion of the attending physician. To improve protocol compliance, an advisory alert tool was developed and integrated in the patient data management system. This alert advises on whether to initiate/continue or stop insulin administration, without giving advice on the dose. Hence, when blood glucose exceeds 215 mg/dl (11.9 mmol/l) on two consecutive measurements with 4-h interval (or one measurement in type 1 diabetics), the alert indicates to administer insulin; when it drops below 180 mg/dl (10 mmol/l), the alert advises to stop insulin administration (or at least taper down in type 1 diabetics). In addition, the alert advises to measure blood glucose again at the latest after 6 h. The nurse/physician is able to overrule the given advice, e.g., to stop insulin when blood glucose concentrations would be above 215 mg/dl (11.9 mmol/l) but the concentrations are dropping rapidly.

### Intervention description {11a}

In the tight blood glucose control group, insulin is administered to target the normal healthy fasting ranges for blood glucose (80–110 mg/dl, 4.4–6.1 mmol/l). Insulin is started as soon as blood glucose exceeds the upper normal limit (110 mg/dl, 4.4 mmol/l). Tight glucose control is guided by the LOGIC-Insulin computerized algorithm [19, 20]. The LOGIC-Insulin software advises the nurse on the insulin dosage (or a dextrose bolus in case of hypoglycemia) as well as on the next blood sampling interval. The software was previously validated in a multicenter trial, demonstrating efficacy and safety [20]. The algorithm takes into account the patient profile, (changes) in nutritional intake, the use of drugs such as steroids, and the trend in blood glucose concentrations and insulin dose. The advised sampling interval varies from 1 to 4 h (and more frequent after hypoglycemia), depending on the (observed and predicted) blood glucose stability. Visual alarms on sampling time, hypoglycemia, and nutrition dose entry errors are included in the software. The software is run from a central server in the hospital onto the client bedside computer. The nurses in charge of the patient operate the program. Because the LOGIC-Insulin software serves as an advising system, the nurse or physician has the ability to overrule the given advice.

### Criteria for discontinuing or modifying allocated interventions {11b}

Tight glucose control is discontinued when the patient starts oral intake of carbohydrates, when the central venous catheter is removed, or at discharge to the general ward or to another ICU not participating in the trial. Eventually, when a patient would stop the oral intake of carbohydrates again while still in ICU, or when the central venous catheter would be replaced in ICU, the intervention is resumed. Upon discontinuation of the study intervention, conventional blood glucose management is applied, which may slightly differ per center, but in general signifies a liberal blood glucose management.

Also in the control group, the alert tool is used until the patient starts oral intake of carbohydrates, when the central venous catheter is removed, or at discharge to the general ward or to another ICU not participating in the trial. At that time, conventional blood glucose management is applied, which may slightly differ per center, but in general signifies a liberal blood glucose management. Eventually, when a patient would stop the oral intake of carbohydrates again while still in ICU, or when the central venous catheter would be replaced in ICU, the alert tool is used again.

### Strategies to improve adherence to interventions {11c}

The compliance to the allocated study protocol is monitored daily by the clinical trial assistants (CTAs). In both groups, adherence to the protocol is facilitated by the use of decision-support software. In the tight glucose control group, the LOGIC-Insulin software is used, which was previously validated and which advises the nurse on the insulin dosage (or a dextrose bolus in case of hypoglycemia) as well as on the next blood sampling interval [20]. In the liberal glucose control group, an advisory alert tool is used that advises on whether to initiate, continue, or stop insulin administration. The alert also advises to measure blood glucose again at the latest after 6 h.

### Relevant concomitant care permitted or prohibited during the trial {11d}

In accordance with the recent feeding guidelines for critically ill patients, enteral nutrition is started as soon as possible [25]. When enteral nutrition is insufficient to meet the caloric requirements, supplemental parenteral nutrition is not initiated before day 8 in ICU, in accordance with recent evidence [17, 18]. Except from a small amount of parenteral glucose, no other macronutrients are administered by the parenteral route before day 8. Only in the most severely malnourished patients (body mass index below 17 kg/m^2^) and in patients readmitted to the ICU, parenteral nutrition can be initiated earlier, as these patients were excluded from large feeding RCTs [17]. In case early parenteral nutrition would be planned upon ICU admission in such patient, the patient is not included in the trial. In general, the amount of parenteral glucose given during the first week in ICU by maintenance solutions may not exceed the equivalent amount of 1 ml dextrose 5% per kg per hour, unless the patient develops spontaneous hypoglycemia (hypoglycemia while not on insulin treatment) or has high risk to do so (e.g., in acute liver failure), or when the patient has a need for high volumes of hypotonic fluids (e.g., severe hypernatremia due to fluid losses). Until the patient receives at least 80% of his/her caloric needs of enteral nutrition, micronutrients (trace elements, minerals and vitamins) are administered parenterally to prevent refeeding syndrome, according to local standard practice.

### Provisions for post-trial care {30}

When the RCT would show efficacy and safety of one of two studied treatment strategies, this treatment strategy will be applied to all ICU patients of participating centers.

In accordance with the Belgian and European legislation, the sponsor has a no fault insurance that covers any damage incurred by a study patient and linked directly or indirectly to the participation to the study.

### Outcomes {12}

To provide insight in the quality of glucose control in both groups, blood glucose metrics in ICU will be reported, as mentioned in the protocol (see Additional file 1), such as the peak and mean glucose concentrations in ICU, and the incidence of moderate (40–70 mg/dl, 2.2–3.9 mmol/l) and severe (<40 mg/dl; <2.2 mmol/l) hypoglycemia during ICU stay.

The primary outcome is the duration of dependency on intensive care. The duration of dependency on intensive care will be reported as the crude number of ICU-stay days and as the time to live discharge from ICU, to account for mortality as competing risk. ICU non-survivors will be censored beyond the longest duration of ICU length of stay of the survivors. As the timing of ICU discharge to a regular ward may be affected by the availability of beds on regular wards, which could induce bias, we decided to analyze “time to discharge from ICU” as “time to ready for discharge from ICU.” A patient is considered “ready for discharge” as soon as all clinical conditions for ICU discharge have been fulfilled (no longer in need for, or at risk of, vital organ support).

Safety endpoints include:

- Hospital mortality
- Mortality in ICU
- Mortality 90 days post randomization
- Incidence of severe hypoglycemia resistant to intravenous glucose administration

Secondary efficacy endpoints include:

- Hospital length of stay and time to (live) discharge from hospital
- Time to final (live) weaning from mechanical respiratory support and the need for tracheostomy
- The incidence and type of new infections during ICU stay, and the duration of antibiotic treatment in ICU
- Markers of inflammation, including peak values and time profiles
- Presence of clinical, electrophysiological, morphological, and molecular signs of respiratory and peripheral muscle weakness during ICU stay
- New kidney injury during ICU stay: the presence or absence and duration of new kidney injury during ICU according to KDIGO criteria (Kidney Disease: Improving Global Outcomes) [26, 27]; proportion of patients in need of new initiation of renal replacement therapy in ICU; duration of renal replacement therapy in ICU; recovery from kidney injury and from renal replacement therapy.
- The need for pharmacological or mechanical hemodynamic support during ICU stay and its duration, and the time to final (live) weaning from all pharmacological or mechanical support
- The incidence and recurrence of atrial fibrillation during ICU stay, duration of atrial fibrillation, number of episodes of atrial fibrillation and treatment for atrial fibrillation (in selected centers)
- The incidence of major adverse cardiovascular events (non-fatal myocardial infarction, non-fatal stroke, low cardiac output syndrome, and cardiovascular death) during ICU stay (in selected centers)
- The time course of markers of liver dysfunction in ICU, including markers of cholestatic and cytolytic liver dysfunction
- The number of readmissions to the ICU within 48 h after discharge
- The presence or absence of delirium during ICU stay (in selected centers)
- Long-term functional outcome:
  - ◦ For all patients: a validated health questionnaire (Short Form 36, SF-36) 2 years ± 2 months after inclusion.
  - ◦ Subgroup of brain-injured patients (i.e., patients admitted because of traumatic brain injury, subarachnoid hemorrhage, intracranial bleeding, ischemic stroke, or out-of-hospital cardiac arrest): additional functional outcome after 6(±1) and 12(±1) months (extended Glasgow outcome scale and modified Rankin scale)
- Use of intensive care resources (costs for hospitalization, for honoraria for medical and allied healthcare services, for pharmacy, for blood products, for clinical chemistry, for radiology, for graft products and for other expenses)

Depending on additional funding, further preplanned studies, of which the detailed protocols will be reported separately, include:

- Muscle strength, rehabilitation, recovery of organ function and survival up to 4 years post randomization
- Mechanistic studies. The effect of the intervention will be studied in relation to outcome on biochemical, metabolic, immunological, endocrine, inflammatory, coagulation, cardiac and (epi)genetic markers on blood, pleural/pericardial fluid, urine and tissue samples up to 4 years post randomization. Markers include, among others, glucose, lipid, ketone and amino acid concentrations, cytokines, hypothalamic-pituitary hormones, glucagon, and C-peptide.

### Participant timeline {13}

**Table:** 

	Preoperative visit	ICU admission	Day 1 till ICU discharge	ICU discharge	Beyond ICU discharge
Informed consent^a^	X	X			
Randomization and start of study intervention		X			
Measurement of blood glucose and titration of insulin according to the allocated intervention		X	X		
Blood and urine sample^b^		X	X		
Clinical assessment of muscle weakness and ICU functional status^c^			X		
Electrophysiological and morphological assessment of muscle weakness, and needle biopsy^d^			X		
Discontinue study intervention^e^				X	
Assessment of long-term survival and functional status					X

^a^Depending on whether the admission is elective or urgent, and whether one is able to contact the patient before ICU admission, informed consent is obtained prior or shortly after ICU admission (deferred informed consent)

^b^Blood and urine samples are taken as per routine practice. Routine daily measurements include routine clinical chemistry, hematology, and markers of inflammation. Other routine measurements are only determined on selected days, as per local practice. In selected centers, extra biological samples are taken upon admission (blood samples) and thereafter daily (blood and urine samples) for research purposes. In selected centers, samples of pericardial and pleural fluid are collected upon admission and thereafter daily, in patients admitted after cardiac surgery, as described in the protocol

^c^These tests are performed on selected days in ICU, as described in the protocol

^d^This is performed on selected days in ICU (in selected centers, depending on additional funding), as described in the protocol

^e^The study intervention is discontinued upon ICU discharge or earlier, in case the patient is able to resume oral feeding or when the central venous catheter is removed and not replaced

### Sample size {14}

To detect a reduction in ICU dependency by 1 day with at least 80% power (two-tailed) and 95% certainty, and assuming a baseline mean ICU stay of 9 days and a standard deviation of 15, 2782 patients in each group need to be included (total 5564). For safety reasons, we also want to exclude any clinically relevant harmful impact on hospital mortality (safety endpoint). To detect an adversely increased hospital mortality from 8.5 to 10% with 80% power and 95% certainty, 4612 patients per group need to be included (total 9224). Hence, we plan to include 9230 patients.

The baseline mean ICU stay and its standard deviation are based on our previous multicenter RCT studying the impact of early parenteral nutrition, which was performed in the same or comparable centers in the same healthcare system [17]. As the baseline ICU stay may have changed over time and may differ per center, and as it is difficult to predict the relative contribution of each center, an interim analysis was planned after inclusion of 25% of the study population (*n*=2308), to allow adjustment of sample size if needed, based on the observed data in the control group. At that time, the independent data monitoring committee indicated that there was no need to adjust the sample size.

### Recruitment {15}

All patients admitted to one of the participating ICUs are screened for eligibility upon ICU admission (within 2 h).

## Assignment of interventions: allocation

### Sequence generation {16a}

Consecutive patients are randomly assigned to one of the treatment groups using a centralized computer randomization stratified by center and diagnostic category upon ICU admission. Randomization is done in a 1:1 ratio in permuted blocks of 10, and bedside nurses and physicians are unaware of the block size. Diagnostic categories upon ICU admission include:

I Medical ICU admission (infectious or non-infectious): (a) respiratory, (b) cardiovascular, (c) renal, (d) hematological/oncological, (e) gastro-intestinal/hepatic, (f) neurological, (g) metabolic, (h) other
II Surgical/trauma ICU admission: (elective or urgent) surgery and complications after (a) cardiac surgery excluding solid organ transplantation (SOT), (b) thoracic surgery excluding SOT, (c) vascular surgery, (d) abdominal and pelvic surgery excluding SOT, (e) neurosurgery, (f) trauma and burns, (g) solid organ transplantation, (h) neurosurgery

### Concealment mechanism {16b}

Treatment allocation by a centralized computer randomization is done after assessment of the patient for eligibility by the attending physician. Patients are allocated in the order of ICU admission and are assessed for eligibility within the time frame of 2 h of ICU admission, which ensures allocation concealment.

### Implementation {16c}

After assessing eligibility by the treating physician, the computerized randomization program is completed, after which the treatment allocation strategy is revealed. The assigned treatment strategy is communicated with the bedside nurse and ordered in the patient data management system by the treating physician.

## Assignment of interventions: blinding

### Who will be blinded {17a}

Due to the nature of the study, blinding bedside physicians and nurses is not possible. Outcome assessors are blinded, however, as are study participants.

End-of-care decisions in patients for whom further intensive care is considered to be futile will be taken in consensus according to local practice, preferably by a group of at least two senior ICU physicians and the referring specialist, the latter blinded to study treatment allocation.

### Procedure for unblinding if needed {17b}

n/a

Since bedside physicians and nurses are not blinded, there is no need for unblinding.

## Data collection and management

### Plans for assessment and collection of outcomes {18a}

Data are collected daily by experienced and trained CTAs and supported by trained investigators. Clinical data are retrieved from the patient’s electronic health record. Part of the data, including laboratory data and medication administered in ICU, are electronically imported in the eCRF, followed by extensive quality checks. Post-discharge mortality data are available from the National Registry. When this information is not available, vital status is checked through the hospital information system or the regional network of hospital physicians and general practitioners. The assessment of muscle force is performed by trained professionals according to a standard protocol used in all centers. Long-term follow-up includes previously validated scores, as described in the protocol.

### Plans to promote participant retention and complete follow-up {18b}

When consent is withdrawn during ICU stay, the study intervention is stopped, after which the patient receives standard treatment. The patient or patient representative is asked whether or not data collection can be continued without the study intervention. Inherent to the nature of the study (in-ICU intervention), loss of follow-up for the primary endpoint (duration of ICU dependency) and safety endpoint (hospital mortality) is expected to be minimal.

### Data management {19}

The data are collected electronically in a coded electronic case record form (eCRF), unambiguously linked to the source file. Data are manually or semi-automatically transferred and checked for accuracy into the eCRF by the CTA team on a daily basis. Routine laboratory results are imported electronically. Extensive range and consistency checks are regularly performed by the database manager and the study monitor. All original records, such as consent forms and relevant correspondence, are archived at the participating centers, according to local regulations.

### Confidentiality {27}

The collection and processing of data is in accordance with the European General Data Protection Regulation (GDPR) and with the Belgian legislation on the protection of natural persons with regard to the processing of personal data.

The database is user ID/password protected, with logged access control set at network, directory, and database level. The database is stored on secure servers within UZ/KU Leuven and maximally protected by firewalls and login procedures with daily backups. Data are collected in a coded eCRF. For reasons of data integrity and internal control during data input, the patient name is stored in a separate table linked to the eCRF. However, these data are only accessible to the authorized local research staff and the principal database manager on a login/password base. When the database is finalized, the identity data are blinded from the eCRF and only accessible by the study monitor or his substitute.

The biobank is only accessible to authorized people, with a log record of all sample handlings. Samples are labeled with a unique coded number.

### Plans for collection, laboratory evaluation, and storage of biological specimens for genetic or molecular analysis in this trial/future use {33}

Results from routine laboratory measurements performed by the clinical laboratory are collected, as described in the protocol. In selected centers, biological samples (blood samples, urine, pericardial fluid, pleural fluid, muscle/fat biopsy) are collected. The biobank has standard procedures in place to protect adequate storage. Collected biological samples will be stored at −80°C until further analysis.

## Statistical methods

### Statistical methods for primary and secondary outcomes {20a}

A Consolidated Standards of Reporting Trials (CONSORT) diagram will be reported.

The database will be finalized and input of short-term clinical endpoints to be included in the primary study population will be locked 90 days after inclusion of the last patient. Discrete variables will be summarized by frequencies and percentages. Continuous variables will be summarized by use of either mean and standard deviation (SD) or median and interquartile range as appropriate. Results will be analyzed with the use of chi-square testing, Student’s *t* test, or non-parametric testing, as appropriate. Time-to-event effects will be documented by Kaplan-Meier plots with use of log-rank/Wilcoxon testing; the time-to-event effect size will be estimated with the use of Cox proportional hazard analysis. All time-to-event analyses will also be performed on data censored at 90 days. As death is a competing risk for duration of care outcomes, non-survivors will be censored beyond the longest duration of such care required for survivors, as previously reported [17, 18].

Outcomes will be analyzed in an uncorrected manner as well as adjusted for baseline risk factors (including type and severity of illness, age, gender, body mass index, comorbidities including diabetes, center) and will be analyzed with and without censoring at 90 days. For these analyses, *P*-values will be considered significant when at or below 0.05 without correction for multiple testing.

### Interim analyses {21b}

The independent data safety monitoring board (DSMB) performed an interim analysis for safety after inclusion of 50% of the study population. According to the DSMB charter, any recommendation to discontinue the study prematurely should only be based on safety concerns, and not on futility. Stopping boundaries were predefined in the DSMB charter. First, the crude hospital mortality is analyzed by the DSMB statistician blinded to treatment allocation. If the hospital mortality significantly differs (with a *P* value less than 0.017), adjusted hospital mortality is checked. If adjusted hospital mortality (adjusted for baseline risk factors) also differs significantly (with a *P* value less than 0.017), the randomization groups are unblinded, and the DSMB considers a recommendation to stop further recruitment if the data show clear harm by the intervention. The results of the interim analysis and the minutes of the DSMB meetings are confidential within the DSMB.

According to the DSMB charter, an ad hoc meeting of the DSMB may be called at any time by the principal investigators or the DSMB if imminent participants’ safety issues would arise.

As the DSMB is an advisory body, the trial steering committee will have primacy. In the unlikely event that the trial steering group would not accept the recommendation of the DSMB to discontinue the study, the ethical committee will be informed and a meeting with the DSMB will be planned.

After inclusion of 50% of the study population, the DSMB recommended to continue the study as planned.

### Methods for additional analyses (e.g., subgroup analyses) {20b}

To assess whether any impact of the intervention on outcome would be affected by the baseline risk factor subgroup, interaction *P*-values will be calculated using multivariable regression models, with a threshold for significance of interaction set at a *P*-value of <0.1.

The a priori defined subgroups are patients after cardiac surgery as compared with all other patients, patients admitted after elective surgery as compared with all other patients, patients admitted after surgery as compared with all other patients, patients with and without sepsis upon admission, patients with or without a known history of diabetes and/or an elevated HbA1c upon admission, patients admitted for a neurological/neurosurgical reason as compared with all other patients, patients admitted with brain injury as compared with all other patients, patients with high severity of illness score upon admission versus all other patients, and patients with an upon ICU admission predicted short ICU stay versus all other patients.

Since the effect of the intervention may depend on the nutritional strategy [14], which evolves over time in ICU, we will study whether there is a significant interaction between classification into a predicted short ICU stay (with discharge alive from ICU) and the effect of the intervention on outcome. Indeed, whereas patients are relatively starved in the first week in ICU, full feeding including supplemental parenteral nutrition is provided after the first week. However, since the duration of ICU stay may be affected by randomization, we will study the interaction with predicted short ICU stay (with alive ICU discharge) versus predicted prolonged ICU stay (or early ICU mortality). To that purpose, we will develop a statistical model to predict upon ICU admission whether patients will be discharged alive in the first week after ICU admission, or not. The prediction model will be developed in a subgroup of the control arm of the trial. After development of the model, we will study the interaction between classification into predicted short ICU stay with alive discharge and the effect of the intervention on outcome. If the interaction is significant at the 0.1 level, we will study the impact of the intervention on outcome in the subgroups separately. In that case, we will also develop a model to predict which patients have a high chance of either long ICU stay (minimum 10 days in ICU) or of mortality in ICU, to study the impact of the intervention in this subgroup separately.

Finally, we will study whether there is an interaction with observed ICU length of stay (>3 days post randomization versus shorter) in determining the impact of the intervention on outcome.

### Methods in analysis to handle protocol non-adherence and any statistical methods to handle missing data {20c}

All analyses will be done on intention to treat basis. In case of consent withdrawal for further participation in the study, the participant or participant representative is asked whether or not data collection can be continued after stopping the study intervention. In any case, all data that are already collected will be analyzed.

Since the primary and most secondary endpoints are taking place during ICU stay, the majority of data will be available. Of the secondary endpoints, some laboratory measurements may be missing. Missing data for one time point may be interpolated, by calculating the mean of the two neighbor measurements. In other cases, missing data will not be imputed.

### Plans to give access to the full protocol, participant-level data, and statistical code {31c}

Data sharing will be considered only on a collaborative basis, after evaluation of the study protocol and the statistical analysis plan.

## Oversight and monitoring

### Composition of the coordinating center and trial steering committee {5d}

The trial steering committee is responsible for the conduct of the TGC-fast RCT, whereby every participating center is represented. In every center, a lead investigator per unit is responsible for the training of bedside physicians and nurses, and for the training and supervision of CTAs. The CTA team guarantees in every center the daily follow-up of patient screening and inclusion, protocol compliance, collection of outcome data, and the correct storage of biological samples. In the coordinating center, the database manager is responsible for maintenance of the database, and for performing data quality checks. Information technology (IT) specialists are responsible for maintenance of the LOGIC-Insulin software and patient data management system. The study monitor monitors protocol compliance and data collection in accordance with good clinical practice. Regular meetings are organized with the principal investigators and CTAs to discuss the progress of the RCT.

### Composition of the data monitoring committee, its role and reporting structure {21a}

A DSMB has been established to assess the safety of the intervention during and to monitor the overall conduct of the clinical trial. The DSMB consists of three members, including two clinicians experienced in clinical trials and the subject manner, and one statistician. The members are independent of the trial.

Intermittently, the DSMB reviews the trial’s progress by evaluating updated figures on recruitment, data quality, and safety. Specifically, this includes to monitor recruitment rate; to monitor protocol compliance; to monitor the incidence of severe hypoglycemia and the incidence of severe hypoglycemia resistant to intravenous glucose administration in the control and intervention group; to decide whether to recommend that the trial continues to recruit participants or whether recruitment should be terminated, for safety reasons only, based on predefined stopping rules (interim analysis planned after including 50% of the study population); and to monitor the assumptions used to calculate sample size and adjust sample size if necessary to ensure sufficient statistical power (after including 25% of the study population)

The DSMB reports its recommendations to the chair of the trial steering committee. Unless the DSMB would advise the trial steering committee to stop further recruitment because of safety concerns, the trial steering committee remains ignorant of the interim results.

Two interim analyses were planned a priori: a first after including 25% of the study population, to enable repowering; a second after including 50% of the study population to assess safety of the intervention, to allow premature stopping in case the intervention would increase hospital mortality based on predefined stopping criteria. An ad hoc meeting of the DSMB may be called at any time by the principal investigators or the DSMB if imminent participants’ safety issues would arise.

The roles and responsibilities of the DSMB, including the timings of the meetings, the decision-making process, and relationship with other committees, were written in a DSMB charter, based on the recommendations of the DAMOCLES study group [28]. The charter can be provided upon motivated request.

### Adverse event reporting and harms {22}

Critical illness is a condition with adverse outcomes that are expected to occur. In this study, adverse clinical outcomes are listed primary, secondary, or safety endpoints. Hence, none of these study outcome endpoints are considered as serious adverse events or suspected unexpected serious adverse reactions. Any other and unexpected adverse reaction is reported to the study sponsor. For safety reasons, an interim analysis of hospital mortality was performed by the DSMB after inclusion of 50% of the study population to allow early study termination if the intervention group would appear clearly inferior, based on predefined stopping criteria.

The main adverse event of the intervention is hypoglycemia, which warrants prompt treatment. However, ICU physicians and nurses are familiar with the effects of insulin administration and follow-up of blood glucose concentrations. However, severe hypoglycemia (<40 mg/dl, <2.2 mmol/l) that is resistant to intravenous glucose administration is considered as a serious adverse event. This adverse event will be reported to the sponsor, after first knowledge. The immediate report will be followed by detailed, written reports. The sponsor will keep detailed records of all reported adverse events. These records will be submitted to the competent authorities when requested.

### Frequency and plans for auditing trial conduct {23}

The sponsor appointed a monitor, who verifies that the RCT is performed in accordance to the protocol. To that purpose, the sponsor and principal investigators provide direct access to the eCRF, source data, and study master file for monitoring. The monitor also performs extensive range and consistency checks on the collected data.

### Plans for communicating important protocol amendments to relevant parties (e.g., trial participants, ethical committees) {25}

In case of substantial modifications to the protocol, an amendment will be submitted to the ethical committee and the competent authorities. Substantial modifications include modifications that may affect the safety or integrity of the study participants, or the scientific value of the trial. In case of substantial modifications that would impact on ongoing or future treatment, participants will be informed.

### Dissemination plans {31a}

The results of the RCT will be published in a peer-reviewed scientific journal, regardless of the effect size and the direction of the effect (if any). After publication, a link to the study report will be added on the respective trial registry platforms.

## Discussion

The ideal blood glucose target for critically ill patients remains debated, in view of the opposing effects on short-term mortality of previous RCTs [21, 22]. It currently remains unclear whether tight glucose control is effective and safe in the absence of early parenteral nutrition, when provided with an accurate and validated protocol that avoids large glucose fluctuations and hypoglycemia [14]. To this purpose, the TGC-fast RCT randomizes adult critically ill patients to tight versus liberal blood glucose control. In the tight glucose control group, glucose control is guided by the validated LOGIC-Insulin algorithm, which was shown to lead to effective and high-quality glucose control, with a very low incidence of hypoglycemia [19, 20]. Also in the liberal glucose control group, software alerts are implemented in the patient data management system to maximize protocol compliance.

The RCT not only will study short-term clinical outcomes, but also long-term functional outcome and mortality, and biological samples are collected to study potential mechanisms of organ protection or harm (if any). Moreover, the large sample size allows detailed subgroup analyses, since observational studies suggest that the impact of the intervention may be different according to the admission diagnosis and illness severity, and the presence of diabetes [15, 29, 30]. If the intervention shows benefit of the intervention, an effect on healthcare costs will be performed to study whether the intervention is cost-effective.

Regardless of the results of the RCT, the study will inform clinicians on the ideal glucose control strategy. If tight glucose control is confirmed to be beneficial, the strategy can be implemented and guided by the validated LOGIC-Insulin software in comparable adult critically ill patients. If tight glucose control is harmful or not beneficial, the intervention can be abandoned in such patients, which would also lower workload and costs by lowering the need for glucose measurements.

## Trial status

The first patient was included on September 18, 2018. In the first wave of the COVID-19 pandemic, a temporary stop of inclusions in the TGC-fast RCT was mandated by the central ethical committee (from March 15, 2020, till June 8, 2020). After including 25% of the study population, the DSMB indicated that there was no need to adjust the sample size, based on data in the liberal glucose control group. At the safety interim analysis after including 50% of the study population, the DSMB advised the continuation of the study. Recruitment is expected to be completed in the second half of 2022. The short-term clinical outcome data will be analyzed only 90 days after inclusion of the last patient, after locking the database for short-term clinical outcomes.

## Supplementary Information

**Additional file 1.**

## Data Availability

For the primary study publication, the principal investigators will have access to the full dataset. For secondary analyses and studies on collected biological samples, the principal investigators will have access to the dataset necessary for the respective study question. Data sharing will be considered only on a collaborative basis with principal investigators, after evaluation of the proposed study protocol and statistical analysis plan.

## Abbreviations

APACHE-II: Acute Physiology and Chronic Health Evaluation-II
CONSORT: Consolidated Standards of Reporting Trials
CTA: Clinical trial assistant
DSMB: Data safety monitoring board
eCRF: Electronic case record form
GDPR: General Data Protection Regulation
ICU: Intensive care unit
IT: Information technology
KDIGO: Kidney Disease: Improving Global Outcomes
RCT: Randomized controlled trial
SF-36: Short Form 36
SOT: Solid organ transplantation
