# Supplementary material for: Impact of tight blood glucose control within normal fasting ranges with insulin titration prescribed by the Leuven algorithm in adult critically ill patients: the TGC-fast randomized controlled trial
Source: Trials. 2022 Sep 19;23:788. doi: 10.1186/s13063-022-06709-8 (PMC9483886; doi:10.1186/s13063-022-06709-8)
Supplement: Supplementary file 1 — Additional file 1. [file 13063_2022_6709_MOESM1_ESM.zip › TGC-fast protocol version 1.5.pdf]

Impact of Tight blood Glucose Control within normal fasting ranges with insulin titration prescribed by the Leuven algorithm in adult critically ill patients

Study protocol

Protocol Acronym/short title: TGC-fast

Version and date of final protocol:
Version 1.5, January 12, 2021

Trial identifiers
EudraCT Number: 2018-000756-17

Sponsor:
Name: KU Leuven / UZ Leuven
Address: Herestraat 49, 3000 Leuven

Principal Investigator:
Name: Prof. Dr. Greet Van den Berghe
Address: Herestraat 49, 3000 Leuven
Telephone: +32 16 344021
Fax: +32 16 344015
Email: greet.vandenberghe@kuleuven.be

Sub-investigators:
Name: Prof. Dr. Jan Gunst
Address: Herestraat 49, 3000 Leuven
Telephone: +32 16 344021
Fax: +32 16 344015
Email: jan.gunst@kuleuven.be

Name: Prof. Dr. Greet Hermans
Address: Herestraat 49, 3000 Leuven
Telephone: +32 16 344275
Fax: +32 16 34423074
Email: greet.hermans@uzleuven.be

Name: Prof. Dr. Dominique Benoit
Address: De Pintelaan 185, 9000 Gent
Telephone: +32 9 3322775
Fax: +32 9 3324995
Email: Dominique.Benoit@UGent.be

Name: Prof. Dr. Philippe Jorens
Address: Wilrijkstraat 10, 2650 Edegem
Telephone: +32 3 8213635
Fax: +32 3 8284882
Email: Philippe.Jorens@uza.be

Name: Dr. Jasperina Dubois
Address: Stadsomvaart 11, 3500 Hasselt
Telephone: +32 11 308971
Fax: +32 11 211462
Email: jasperina.dubois@jessazh.be

Signatures

______________________________	________________________
Principal Investigator<br>Print Name: Prof. Dr. Greet Van den Berghe	Date
______________________________	________________________
Sub-investigator<br>Print Name: Prof. Dr. Jan Gunst	Date
______________________________	________________________
Sub-investigator<br>Print Name: Prof. Dr. Greet Hermans	Date

Sub-investigator
Print Name: Prof. Dr. Dominique Benoit

Date

Sub-investigator
Print Name: Prof. Dr. Philippe Jorens

Date

Sub-investigator
Print Name: Dr. Jasperina Dubois

Date

Table of Contents

1. Study Synopsis 6
2. Background and rationale 10
3. Trial objectives and Design 12
   - 3.1 Trial objectives 12
   - 3.2 Primary endpoint 12
   - 3.3 Secondary endpoints 13
   - 3.4 Trial Design 15
   - 3.5 Study diagram 16
   - 3.6 Trial Flowchart 17
4. Trial Medication 18
   - 4.1 Investigational Medicinal product and dosing regimen 18
   - 4.2 Drug accountability 18
   - 4.3 Subject compliance 18
   - 4.4 Concomitant medication (non-IMP) 18
5. Selection and withdrawal of subjects 18
   - 5.1 Inclusion criteria 18
   - 5.2 Exclusion criteria 19
   - 5.3 Selection of participants 19
   - 5.4 Randomisation procedure/Code Break 19
   - 5.5 Withdrawal of subjects 20
   - 5.6 Expected duration of trial 20
6. Trial Procedures 20
   - 6.1 Randomized intervention 20
   - *6.1.1 Tight blood glucose control* 21
   - *6.1.2 Liberal blood glucose control* 22
   - 6.2 Common strategy for glucose measurement, insulin administration and feeding 23
   - 6.3 Laboratory tests 23
   - 6.4 Other investigations 25
7. Assessment of efficacy 28

8. Assessment of Safety 28
8.1 Notification of adverse events and serious adverse events 28
8.2 Treatment stopping rules 29
8.3 Data monitoring committee (DMC) 29
9. Statistics 30
9.1 Sample size 30
9.2 Randomisation 31
9.3 Analysis 31
10. Quality assurance 32
11. Direct access to source data and documents 32
12. Ethics and regulatory approvals 32
13. Data Handling 33
14. Data Management 40
15. Translational research 41
16. Publication Policy 41
17. Insurance/Indemnity 41
18. Financial Aspects 41
19. Appendices 41
20. References 41

1. Study synopsis

Title of clinical trial	Impact of Tight blood Glucose Control within normal fasting ranges with insulin titration prescribed by the Leuven algorithm in adult critically ill patients
Protocol Short Title/Acronym	TGC-fast
Study Phase if not mentioned in title	Phase 3
Sponsor name	KU Leuven / UZ Leuven
Principal Investigator	Leading principal investigator:<br>Prof. Dr. Greet Van den Berghe<br><br>Principal investigators for KU Leuven/UZ Leuven:<br>Prof. Dr. Jan Gunst<br>Prof. Dr. Greet Hermans<br><br>Principal investigator for the University of Ghent:<br>Prof. Dr. Dominique Benoit<br><br>Principal investigator for the University of Antwerp:<br>Prof. Dr. Philippe Jorens<br><br>Principal investigator for the Jessa Hospital Hasselt:<br>Dr; Jasperina Dubois
Eudract number	2018-000756-17
Medical condition or disease under investigation	Hyperglycemia in adult critically ill patients
Purpose of clinical trial	To investigate whether strictly targeting normal fasting ranges for blood glucose with use of a validated algorithm is clinically superior to tolerating hyperglycemia in adult critically ill patients in the context of withholding parenteral nutrition during the first week in the intensive care unit (ICU)
Primary objective	To study the short-term clinical impact of the studied intervention (short-term morbidity and mortality)
Secondary objectives	- To study the long-term impact of the intervention (morbidity and mortality)

	- To study the economic impact (healthcare resources)<br>- To study the pathophysiological mechanisms involved (depending on additional funding)
Trial Design	Multicenter, open label, randomized, parallel group efficacy, safety and tolerability study
Endpoints	Primary endpoints:<br>The duration of ICU dependency (defined as the crude number of days with need for vital organ support and as the time to live discharge from ICU to account for death as competing risk)<br><br>Secondary endpoints:<br>- Glucose metrics in ICU, with and without censoring at 90 days (mean/median morning blood glucose concentration, mean daily blood glucose concentration, incidence of moderate and severe hypoglycemia during ICU stay, peak blood glucose concentration after hypoglycemic event, duration of hypoglycemia, number of hypoglycemic events per patient, minimum and maximum blood glucose concentration per day, time within blood glucose target range, blood glucose variability, hyperglycemic index)<br>- Mortality in ICU and in hospital, with and without censoring at 90 days<br>- Mortality 90 days post randomization<br>- Hospital length of stay, with and without censoring at 90 days<br>- Time to (live) discharge from hospital, with and without censoring at 90 days<br>- Time to final (live) weaning from mechanical respiratory support, with and without censoring at 90 days<br>- The duration of ICU dependency (defined as the crude number of days with need for vital organ support and as the time to live discharge from ICU to account for death as competing risk), with censoring at 90 days<br>- The incidence of new infections during ICU stay, with and without censoring at 90 days<br>- The need for tracheostomy during ICU stay, with and without censoring at 90 days<br>- Presence of clinical, electrophysiological and morphological signs of respiratory and peripheral muscle weakness during ICU

	stay in patient subgroups in selected centers, with and without censoring at 90 days<br>- The presence or absence of new kidney injury during ICU stay, and duration and recovery herefrom, with and without censoring at 90 days<br>- The need for new initiation of renal replacement therapy in ICU (incidence, duration and recovery hereof), with and without censoring at 90 days<br>- The need for hemodynamic support during ICU stay, its duration and the time to (live) weaning from hemodynamic support, with and without censoring at 90 days<br>- The incidence of new-onset atrial fibrillation and recurrence of pre-existing atrial fibrillation during ICU stay in selected centers, with and without censoring at 90 days<br>- The incidence of major adverse cardiovascular events (MACE; non-fatal myocardial infarction, non-fatal stroke, low cardiac output syndrome, and cardiovascular death) during ICU stay in selected centers, with and without censoring at 90 days<br>- The presence or absence of signs of liver dysfunction in ICU, with and without censoring at 90 days<br>- The duration of antibiotic treatment during ICU stay, with and without censoring at 90 days<br>- The incidence of bacteremia, and of airway, urinary tract, wound and other infections acquired during ICU stay, with and without censoring at 90 days<br>- Peak and time profile of C-reactive protein concentrations during ICU stay, with and without censoring at 90 days<br>- The number of readmissions to the ICU within 48 hours after discharge, with and without censoring at 90 days<br>- The presence or absence of delirium during ICU stay (in selected centers), with and without censoring at 90 days<br>- Biochemical, metabolic, immunological, inflammatory, cardiac and (epi)genetic markers on blood and tissue samples up to 4 years post randomization (depending on additional funding and in selected centers)<br>- Muscle strength, rehabilitation, recovery of organ function and survival up to 4 years post randomization in selected centers and subgroups of patients (depending on additional funding)<br>- Long-term functional outcome:

	○ For all patients: a validated health questionnaire (Short Form 36, SF-36) 2 years after inclusion<br>○ Subgroup of brain-injured patients: additional functional outcome after 6 and 12 months (extended Glasgow outcome scale and/or modified Rankin scale)<br>- Use of intensive care resources (costs for hospitalization, for honoraria for medical and allied healthcare services, for pharmacy, for blood products, for clinical chemistry, for radiology, for graft products and for other expenses)
Sample Size	9230
Summary of eligibility criteria	Inclusion criteria:<br>All adult (≥18 years of age) patients admitted to one of the participating intensive care units<br><br>Exclusion criteria:<br>- Patients with a DNR (do not resuscitate) order at the time of ICU admission<br>- Patients expected to die within 12 hours after ICU admission (= moribund patients)<br>- Patients able to receive oral feeding (not critically ill)<br>- Patients without arterial and without central venous line and without imminent need to place it as part of ICU management (not critically ill)<br>- Patients previously included in the trial (when readmission is within 48 hours post ICU discharge, the trial intervention will be resumed)<br>- Inclusion in an IMP-RCT of which the PI indicates that co-inclusion specifically in TGC-fast is prohibited<br>- Patients transferred from a non-participating ICU with a pre-admission ICU stay >7 days<br>- Patients planned to receive parenteral nutrition during the first week in ICU<br>- Patients suffering from diabetic ketoacidotic or hyperosmolar coma on ICU admission<br>- Patients with inborn metabolic diseases<br>- Patients with insulinoma<br>- Patients known to be pregnant or lactating<br>- Informed consent refusal

IMP, dosage and route of administration	Insulin through continuous intravenous infusion to target normal fasting blood glucose concentrations (80-110 mg/dl) with use of the LOGIC-insulin algorithm. The insulin infusion rate is adapted according to frequent measurement of blood glucose concentrations advised through the LOGIC-insulin algorithm.
Active comparator product	No insulin except when blood glucose concentrations exceed 215 mg/dl upon two consecutive measurements with 4 hour interval, in which case intravenous insulin infusion is titrated to target blood glucose concentrations between 180 and 215 mg/dl and is stopped as soon as blood glucose falls below 180 mg/dl. In type 1 diabetics, insulin will be initiated after the first measurement above 215 mg/dl and will be titrated to target blood glucose concentrations between 180 and 215 mg/dl.
Maximum duration of treatment of a subject	The intervention is stopped upon ICU discharge, or until the patient is able to resume oral feeding, or until the patient no longer has a central venous catheter, whatever comes first.
Version and date of final protocol	Version 1.5, January 12, 2021
Version and date of protocol amendments	Version 1.5, January 12, 2021.

2. Background and rationale

Critical illness is a life-threatening medical condition that is induced by severe medical illnesses, major trauma or extensive surgeries and is characterized by a continuous dependency on mechanical and/or pharmacological vital organ support, without which death would ensue. Thanks to advances in intensive care medicine, patients now usually survive such conditions that were previously lethal. However, in spite of this progress, about 25% of patients do not recover swiftly from the acute condition. These so-called “prolonged critically ill patients” remain dependent on intensive care for weeks to months. With each day in intensive care, risk of death rises further and survivors have an important long-term legacy (1). The costs for society of intensive care are enormous and predominantly driven by the duration of intensive care stay and by infectious complications (2).

Elevated blood glucose levels (hyperglycemia) are present in virtually every adult patient in the ICU, and the degree of hyperglycemia is related to the risk of adverse outcome (3, 4). Currently, **controversy exists on whether targeting normal blood glucose levels with insulin therapy, referred to as “tight blood glucose control” (TGC), as compared with tolerating hyperglycemia, improves outcome of these patients**. In 3 well designed single center RCTs,

the coordinator (KU Leuven) of this study has demonstrated that morbidity, mortality, use of healthcare resources and long-term functional status significantly improved with TGC (5-10). In particular, the duration of dependency on intensive medical care was reduced substantially. Rapidly, many centers worldwide adopted TGC as part of the standard of care. However, a subsequent large pragmatic multicenter RCT found increased mortality with TGC attributable to more hypoglycemia (low blood glucose levels) (11, 12). Current practice guidelines still recommend some degree of glucose control, but controversy remains about its benefit (13-15) and local practices vary widely in Europe and the rest of the world (16). Irrespective of the degree, blood glucose control is difficult and is time and resource consuming. Two differences between the pioneer studies and the generalizability trial may explain the different results: in the pioneer studies, TGC was performed by expert and extensively trained nurses, which resulted in safer blood glucose control, and early intravenous feeding was used for all patients when enteral feeding was not reaching the caloric goals (table 1). Consortium members have subsequently shown in multicenter RCTs that not using early intravenous feeding improves outcome, with less infectious complications and faster recovery (17, 18). **Hence, it currently remains unknown whether TGC, applied with optimal tools to avoid hypoglycemia, is beneficial without early intravenous feeding.**

Table 1: Comparison of landmark trials on blood glucose control in critically ill patients

	**Pioneer studies (Leuven)**	**NICE-SUGAR**
**Number of patients**	3448	6100
**Population**	Critically ill adults and children	Critically ill adults
**Effect of TGC**	Reduced morbidity<br>Reduced mortality<br>Reduced long-term legacy<br>Reduced healthcare costs	Increased mortality<br>No effect on morbidity
**Strengths**	- Accurate glucose measurements, highly standardized<br>- Standardized protocol by extensively trained nurses, low incidence of hypoglycemia[1]	- Multicenter<br>- Pragmatic, “real life” and thus not standardized context
**Weaknesses**	- Nutritional strategy comprised the use of **early parenteral nutrition**, later shown to be harmful	- **Inaccurate glucose measurements**<br>- Un-validated **“if-then” algorithm**, with <50% of measurements within target and **high incidence of hypoglycemia**

[1] The performance of the protocol has been improved further by the LOGIC-Insulin software.

The **aim of TCG-fast** is to compare the effectiveness of TGC versus tolerating hyperglycemia in adult critically ill patients of both sexes **who are not receiving intravenous nutrition for up to one week in the ICU**. To this purpose, we will perform a multicenter RCT that is adequately powered for patient-centered and economic endpoints. Patients will be randomly allocated to TGC to target normal fasting glucose levels with insulin versus tolerating hyperglycemia up to a predefined level. TGC will be guided by our previously developed computer algorithm that has shown even to outcompete the well-trained nurses, with virtually preventing hypoglycemia (LOGIC-Insulin) (19). The efficacy and safety of the LOGIC-Insulin algorithm has been confirmed in a multicenter context by members of this consortium (20).

3. Trial objectives and design

3.1 Trial objectives

The main objective of this study is to test the hypothesis that in adult critically ill patients receiving an evidence-based, restrictive feeding regimen (no parenteral nutrition in the first week of critical illness), targeting normal blood glucose concentrations (80-110 mg/dl) reduces short-term morbidity and dependency on intensive care as compared with tolerating hyperglycemia up to 215 mg/dl. The null hypothesis is that there will be no difference in the primary outcome measures (incidence of new infections during ICU stay, ICU length of stay and the time to live discharge from ICU) between the two strategies. The study is two-tailed so that it is also powered to detect a negative impact of targeting normal blood glucose concentrations. As a safety endpoint, mortality will be monitored. These analyses will be done unadjusted as well as adjusted for risk factors.
The secondary objectives are:

- To study the long-term impact of the intervention (morbidity and mortality)
- To study the economic impact (healthcare resources)
- To study the underlying mechanisms that may explain an eventual benefit (or harm) from targeting normal blood glucose concentrations with intensive insulin therapy (depending on additional funding). This will be achieved by studying the effects of treatment allocation on metabolic, endocrine, inflammatory, coagulation, cardiac and (epi)genetic markers in blood and tissue samples of critically ill patients and by studying the effects of treatment allocation on the innate immune response and pathways of inflammation and autophagy.

3.2 Primary endpoint

The primary outcome is the duration of dependency on vital organ support and intensive medical care, without censoring at 90 days. The duration of dependency on vital organ support

and intensive medical care will be reported as the crude number of ICU-stay days and as the time to live discharge from ICU, to account for mortality as competing risk. ICU non-survivors will be censored beyond the longest duration of ICU length of stay of the survivors. As the timing of ICU discharge to a regular ward may be affected by the availability of beds on regular wards, which could induce bias, we decided to analyze ‘time to discharge from ICU’ as ‘time to *ready for* discharge from ICU’. A patient is considered ‘ready for discharge’ as soon as all clinical conditions for ICU discharge have been fulfilled (no longer in need for, or at risk of, vital organ support).

3.3 Secondary endpoints

- Glucose metrics in ICU: mean and median morning blood glucose concentrations; mean daily blood glucose concentration; incidence of moderate (40-70 mg/dl) and severe (<40 mg/dl) hypoglycemia during ICU stay; peak of blood glucose after correction of hypoglycemia; duration of hypoglycemia; number of hypoglycemic events per patient; minimum and maximum blood glucose concentration per day; time within blood glucose target range; blood glucose variability; hyperglycemic index. These parameters will be studied during ICU stay, with and without censoring at 90 days
- Mortality in ICU and in hospital, with and without censoring at 90 days
- Mortality 90 days post randomization
- Hospital length of stay, with and without censoring at 90 days
- Time to (live) discharge from hospital, with and without censoring at 90 days. Hospital non-survivors will be censored beyond the longest duration of hospital length of stay of the survivors.
- Time to final (live) weaning from mechanical respiratory support, with and without censoring at 90 days. ICU non-survivors will be censored beyond the longest duration of mechanical respiratory support of the survivors.
- The duration of ICU dependency (defined as the crude number of days with need for vital organ support and as the time to live discharge from ICU to account for death as competing risk), with censoring at 90 days
- The incidence of new infections during ICU stay, with and without censoring at 90 days. Two experts in infectious diseases, who will be blinded for study group assignment, will determine, in consensus, the presence of infection on admission to the ICU and the presence of infection acquired after randomization. They will make their decision on the basis of guidelines in the study protocol (see 13. Data handling).
- The need for tracheostomy during ICU stay, with and without censoring at 90 days
- Presence of clinical, electrophysiological and morphological signs of respiratory and peripheral muscle weakness during ICU stay in patient subgroups in selected centers, with and without censoring at 90 days

- New kidney injury during ICU stay, with and without censoring at 90 days: the presence or absence of new kidney injury during ICU according to modified KDIGO criteria (Kidney Disease: Improving Global Outcomes) (21, 22); proportion of patients in need of new initiation of renal replacement therapy in ICU; duration of renal replacement therapy in ICU; recovery from kidney injury
- The need for pharmacological or mechanical hemodynamic support during ICU stay and its duration, with and without censoring at 90 days. In addition, time to final (live) weaning from all pharmacological or mechanical support will be analyzed (with and without censoring at 90 days), with ICU non-survivors censored beyond the longest duration of pharmacological or mechanical hemodynamic support of the survivors.
- The incidence of new-onset atrial fibrillation and recurrence of pre-existing atrial fibrillation during ICU stay in selected centers, with and without censoring at 90 days. In order to quantify and phenotype burden of atrial fibrillation, we will perform a detailed analysis of atrial fibrillation occurrence, including timing of onset, duration and number of episodes. Strategies regarding medical and/or electrical rhythm control, as well as rate control will be documented.
- The incidence of major adverse cardiovascular events (MACE; non-fatal myocardial infarction, non-fatal stroke, low cardiac output syndrome, and cardiovascular death) during ICU stay in selected centers, with and without censoring at 90 days
- The presence or absence of signs of liver dysfunction in ICU, with and without censoring at 90 days: cholestatic liver dysfunction, cytolytic liver dysfunction.
- The duration of antibiotic treatment during ICU stay, with and without censoring at 90 days
- The incidence of bacteremia, airway, urinary tract, wound and other infections (all with and without censoring at 90 days).
- Inflammation: the effect of the intervention on markers of inflammation will be analyzed by comparing the distribution of the highest value of C-reactive protein during ICU stay and changes from baseline to the highest value and by comparing time profiles of daily C-reactive protein values (with and without censoring at 90 days).
- The number of readmissions to the ICU within 48 hours after discharge (with and without censoring at 90 days)
- The presence or absence of delirium during ICU stay (in selected centers), with and without censoring at 90 days. To that purpose, the worst Intensive Care Delirium Screening Checklist (ICSDC) score observed per day in ICU will be entered in the database.
- Biochemical, metabolic, immunological, endocrine, inflammatory, cardiac and (epi)genetic markers on blood and tissue samples up to 4 years post randomization (depending on additional funding and in selected centers)
- Muscle strength, rehabilitation, recovery of organ function and survival up to 4 years post randomization in selected centers and subgroups of patients (depending on additional funding)

- Long-term functional outcome:
  - For all patients: a validated health questionnaire (Short Form 36, SF-36) 2 years ± 2 months after inclusion
  - Subgroup of brain-injured patients (i.e. patients admitted because of traumatic brain injury, subarachnoid hemorrhage, intracranial bleeding, ischemic stroke or out-of-hospital cardiac arrest): additional functional outcome after 6(±1) and 12(±1) months (extended Glasgow outcome scale and/or modified Rankin scale)
- Use of intensive care resources (costs for hospitalization, for honoraria for medical and allied healthcare services, for pharmacy, for blood products, for clinical chemistry, for radiology, for graft products and for other expenses)

3.4 Trial design

The study is a multicenter, open-label, randomized, parallel group efficacy, safety and tolerability study.

3.5 Study diagram

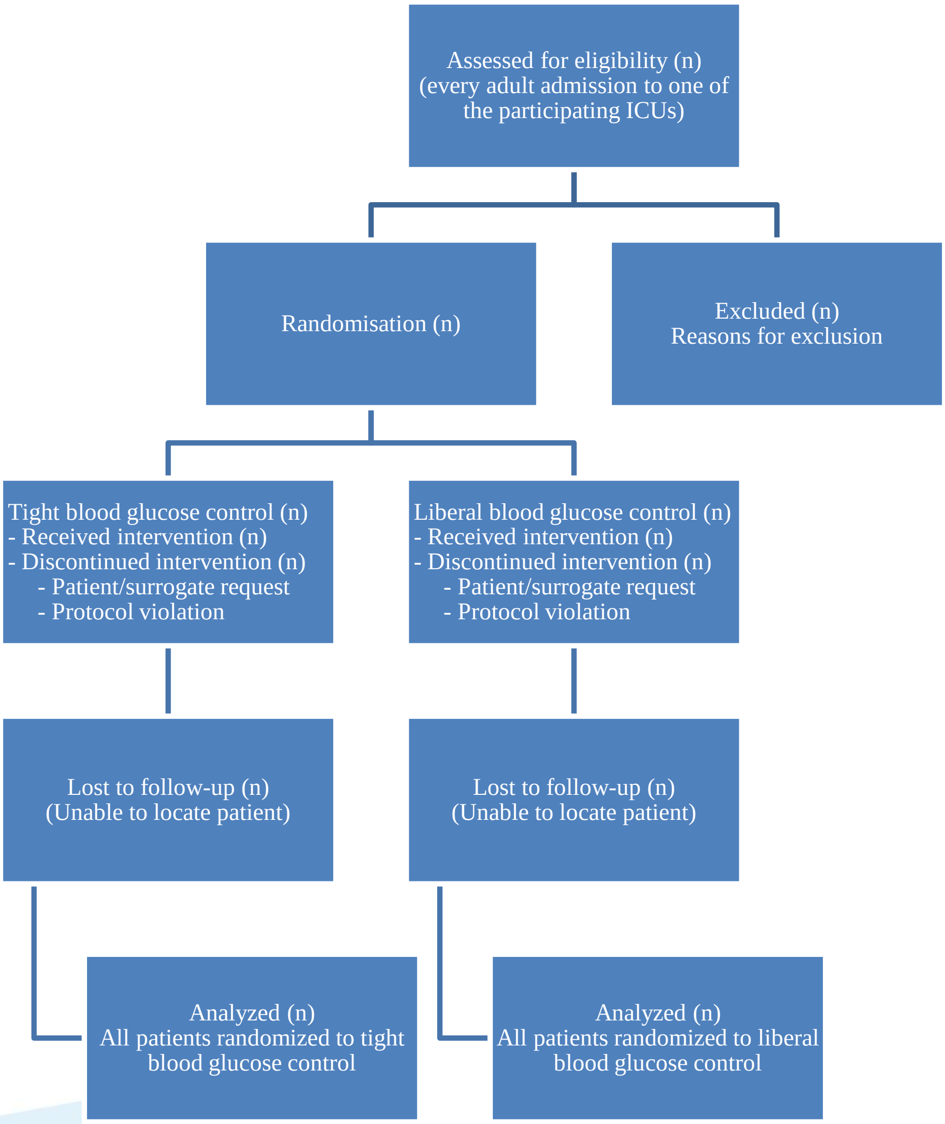

3.6 Trial flowchart

	Preoperative visit	ICU admission	Day 1 till ICU discharge	ICU discharge	From ICU till hospital discharge
Informed consent[1]	X	X			
Randomization and start of study intervention		X			
Measurement of blood glucose (6x/day, can be adjusted according to stability of the profile and as advised by the LOGIC-insulin algorithm)		X	X		
Blood and urine sample[2]		X	X		
Electrophysiological and clinical tests of muscle weakness[3]			X	X	
Tissue samples[4]			X		
Discontinue study intervention[5]				X	

[1] Depending on whether the admission is elective or urgent, and whether one is able to contact the patient before ICU admission, informed consent will be obtained prior or shortly after ICU admission (deferred informed consent).

[2] A blood sample is collected upon admission and thereafter daily. Daily, a urine sample is collected. Routine daily measurements include routine clinical chemistry, hematology and markers of inflammation. Other routine measurements are only determined on selected days. These include liver enzymes and coagulation tests. In selected centers, extra blood and plasma samples, as well as urine samples will be stored for research purposes in order to study selected biochemical, metabolic, immunological, endocrine, inflammatory and (epi)genetic markers.

[3] These tests will be performed on selected days in ICU (in selected centers).

[4] As described in 6.4 and depending on additional funding, in selected centers and subgroups of patients, tissue biopsies will be taken at selected days in ICU.

[5] The study intervention is discontinued upon ICU discharge or earlier, in case the patient is able to resume oral feeding or when the central venous catheter is removed and not replaced.

4. Trial medication

4.1 Investigational medicinal product and dosing regimen

Short-acting insulin will be provided by the hospital pharmacy. Insulin will be dissolved in a 50 ml solution of normal saline and will be administered through continuous intravenous infusion. The concentration will be 1-2 IU/ml. No boluses of insulin will be allowed. Insulin will be infused through a lumen with constant basal flow rate, with avoidance of bolus medication or fluid boluses through that lumen. Insulin should be infused by a syringe pump through a central venous catheter. The three-way-valve through which the syringe pump infusing insulin is connected to the intravenous line, should be as close to the patient as possible. The manufacturer of insulin and the used syringe pump may vary per center. This information will be kept by the sponsor in a separate file. In appendix, the Summary of Product Characteristics of insulin is added.

4.2 Drug accountability

Not applicable.

4.3 Subject compliance

Not applicable.

4.4 Concomitant medication (non-IMP)

There are no restrictions for concomitant medication. As mentioned above, insulin will be administered through a dedicated lumen of a central venous line by continuous intravenous infusion. In order to avoid boluses of insulin with resultant hypoglycemia and/or large fluctuations in blood glucose concentrations, no boluses of (other) medication or fluids will be allowed through that lumen.

5. Selection and withdrawal of subjects

5.1 Inclusion criteria

All adult patients (≥18 years of age) admitted to one of the participating ICUs are eligible for inclusion.

5.2 Exclusion criteria

- Patients with a DNR (do not resuscitate) order at the time of ICU admission. Patients refusing blood transfusion upon ICU admission, will be considered as having a therapy restriction upon admission and will not be included.
- Patients expected to die within 12 hours after ICU admission (= moribund patients)
- Patients able to receive oral feeding (not critically ill)
- Patients without arterial and without central venous line and without imminent need to place it as part of ICU management (not critically ill)
- Patients previously included in the trial (when readmission is within 48 hours post ICU discharge, the trial intervention will be resumed)
- Inclusion in an IMP-RCT of which the PI indicates that co-inclusion specifically in TGC-fast is prohibited
- Patients transferred from a non-participating ICU with a pre-admission ICU stay >7 days
- Patients planned to receive parenteral nutrition during the first week in ICU, including patients in need of a high dose of parenteral glucose to treat spontaneous hypoglycemia due to acute liver failure (minimum 200g intravenous glucose/day) and patients planned to receive peritoneal dialysis in the first week in ICU. The administration of parenteral nutrition before ICU admission is not a contraindication, provided that parenteral nutrition is stopped or planned to be stopped upon ICU admission.
- Patients suffering from diabetic ketoacidotic or hyperosmolar coma on ICU admission
- Patients with inborn metabolic diseases
- Patients with insulinoma
- Patients known to be pregnant or lactating
- Informed consent refusal

5.3 Selection of participants

As mentioned above, all adult patients admitted to one of the participating ICUs will be screened for eligibility during the study period.

5.4 Randomization procedure/code break

The study will use a prospective, randomized, controlled, parallel group design. On admission, patients will be randomly assigned to tight control of blood glucose to healthy fasting ranges (80-110 mg/dl) versus liberal blood glucose control (no insulin except when blood glucose concentrations exceeds 215 mg/dl upon two consecutive measurements with 4 hour interval, in which case insulin intravenous infusion is titrated to target blood glucose between 180 and 215 mg/dl and is stopped as soon as blood glucose falls below 180 mg/dl; in type 1 diabetics, insulin

will be started after the first measurement above 215 mg/dl and will be tapered down when blood glucose falls below 180 mg/dl). At ICU admission, consecutive patients will be randomly assigned to one of these treatment groups using a centralized computer randomization. If a patient cannot be included upon ICU admission because he/she is not critically ill (able to eat, no need for arterial and central venous line), the patient can be included at a later time point when his/her clinical condition would worsen and there are no exclusion criteria anymore. Randomization will be done in a 1:1 ratio in permuted blocks of 10 and stratified according to primary diagnostic category on admission:

I Medical ICU admission (infectious or non-infectious): (a) respiratory, (b) cardiovascular, (c) renal, (d) hematological/oncological, (e) gastro-intestinal/hepatic, (f) neurological, (g) metabolic, (h) other

II Surgical/trauma ICU admission: (elective or urgent) surgery and complications after (a) cardiac surgery excluding solid organ transplantation (SOT), (b) thoracic surgery excluding SOT, (c) vascular surgery, (d) abdominal and pelvic surgery excluding SOT, (e) neurosurgery, (f) trauma and burns, (g) solid organ transplantation, (h) neurosurgery

Bedside nurses and physicians will be unaware of the block size.

5.5 Withdrawal of subjects

When a patient or legal representative withdraws consent during ICU stay, the study intervention will be stopped and patient will receive standard treatment. The patient or legal representative will be asked whether or not data collection can be continued without the study intervention. A separate patient log will register all patients withdrawn from the study.

End-of-care decisions in patients for whom further intensive care is considered to be futile will be taken in consensus according to local practice, preferably by a group of at least two senior ICU physicians and the referring specialist, the latter blinded to study treatment allocation.

5.6 Expected duration of trial

Based on our previous studies, we expect a high inclusion rate. The last inclusion is planned 2 years after inclusion of the first patient. Data interpretation and publication of the short-term outcome will take 12 months.

6. Trial procedures

6.1 Randomized intervention

Written informed consent will be obtained from the patient or his/her closest family member or legal representative. Depending on whether the admission is elective or urgent, and whether one is able to contact the patient before ICU admission, informed consent will be obtained prior to or shortly after ICU admission. For planned ICU admissions after elective procedures, informed consent will be asked prior to the procedure when possible. For urgently admitted patients, obtaining informed consent prior to ICU admission is impossible. As a blood glucose control strategy has to be initiated upon admission, treatment allocation will be done after assessment of the patient for eligibility by the attending physician within the time frame of two hours (deferred informed consent when one was not able to contact the patient or legal representative before ICU admission). In the latter case (deferred informed consent), maximal efforts will be done to obtain a written informed consent form within 72 hours after ICU admission. A duplicate of the signed informed consent will be given to the patient or the closest family member or legal representative. In a considerable number of patients, especially in urgently admitted patients, we expect that informed consent will have to be obtained from the patient's closest family member or legal representative. As regaining consciousness and mental competence often occurs gradually and slowly in these patients, it is very difficult to determine at what time the patient is able to give a valid informed consent. In addition, recovery can take from several days to several months. Often, patients are only able to give a valid consent after discharge from ICU, at what time the intervention is already terminated. Therefore, we will give an opting out form to the legal representative, who can give it to the patient once he/she is well recovered. With this document, the patient has the possibility to terminate the study intervention (if still applicable) and/or withdraw from further data collection. When a patient is readmitted to the ICU within 48 hours after ICU discharge, the trial intervention he/she was assigned to during the first ICU admission will be resumed. Patients readmitted later than 48 hours after the initial ICU discharge will be treated according to local standard practice.

6.1.1 Tight blood glucose control

In the tight blood glucose control group, insulin will be administered to target the normal healthy fasting ranges for blood glucose (80-110 mg/dl, 4.4-6.1 mmol/l). Insulin is started as soon as blood glucose exceeds the upper normal limit (110 mg/dl). TGC will be guided by the LOGIC-Insulin computerized algorithm (19, 20). The LOGIC-Insulin software advises the nurse on the insulin dosage (or a dextrose bolus in case of hypoglycemia) as well as on the next blood sampling interval. The software was previously validated in a multicenter trial, demonstrating efficacy and safety (20). The algorithm takes into account the patient profile, (changes) in nutritional intake, the use of drugs such as steroids and the trend in blood glucose concentrations and insulin dose. The advised sampling interval varies from 1 to 4 h (and more frequent after hypoglycemia), depending on the (observed and predicted) blood glucose

stability. Visual alarms on sampling time, hypoglycemia, and nutrition dose entry errors are included in the software. The software is run from a central server in the hospital onto the client bedside computer. The nurses in charge of the patient operate the program. Because the LOGIC-Insulin software serves as an advising system, the nurse has the ability to overrule the given advice. Overrules are defined as absolute insulin dose differences >0.1 and <1 IU/h for minor overrules and ≥1 IU/h for major overrules. TGC will be discontinued when the patient starts oral intake of carbohydrates, when the central venous catheter is removed, or at discharge to the general ward or to another ICU not participating in the trial. Eventually, when a patient would stop the oral intake of carbohydrates again while still in ICU, or when the central venous catheter would be replaced in ICU, the intervention is resumed. Upon discontinuation of the study intervention, conventional blood glucose management will be applied, which may slightly differ per center, but in general signifies a liberal blood glucose management.

6.1.2 Liberal blood glucose control

In the liberal blood glucose control group, insulin will only be initiated when blood glucose levels exceed 215 mg/dl (11.9 mmol/l) on two consecutive measurements, with the dose adjusted by the nurses/physicians to maintain concentrations between 180 and 215 mg/dl (10-11.9 mmol/l). When blood glucose drops below 180 mg/dl (10 mmol/l), insulin infusion is stopped. In type 1 diabetics, insulin will be initiated after the first blood glucose measurement above 215 mg/dl and the infusion rate will be adjusted to maintain blood glucose concentrations between 180 and 215 mg/dl. Blood glucose is measured minimum 4 times per day. The management of eventual hypoglycemia is at the discretion of the attending physician. To improve protocol compliance, an advisory alert tool was developed. This alert advices on whether to initiate/continue or stop insulin administration, without giving advice on the dose. Hence, when blood glucose exceeds 215 mg/dl on two consecutive measurements with 4 hour interval (or one measurement in type 1 diabetics), the alert indicates to administer insulin, when it drops below 180 mg/dl, the alert advices to stop insulin administration (or at least taper down in type 1 diabetics). In addition, the alert will advise to measure blood glucose again at the latest after 6 hours. The nurse/physician is able to overrule the given advise, e.g. to stop insulin when blood glucose concentrations would be above 215 mg/dl but the concentrations are dropping rapidly. The alert tool will be used until the patient starts oral intake of carbohydrates, when the central venous catheter is removed, or at discharge to the general ward or to another ICU not participating in the trial. Eventually, when a patient would stop the oral intake of carbohydrates again while still in ICU, or when the central venous catheter would be replaced in ICU, the alert tool will be used again. At that time, conventional blood glucose management will be applied, which may slightly differ per center, but in general signifies a liberal blood glucose management.

6.2 Common strategy for glucose measurement, insulin administration and feeding

In both groups, blood glucose concentrations will be measured in undiluted blood, drawn from the arterial line, by an on-site blood gas analyzer. Insulin will be infused through continuous intravenous infusion through a central venous catheter by a syringe pump, usually in concentrations of 50 IU in 50 mL 0.9% NaCl. The concentration can be increased to 100 IU in 50 mL NaCl 0.9% in case of a high insulin need. No boluses of insulin will be allowed. Glucose measurement on arterial blood using an on-site blood gas analyzer yields both a fast and accurate measurement of blood glucose in critically ill patients (23). When the arterial catheter is no longer needed (for medical reasons), blood glucose will be measured on capillary blood using a glucometer with a validated performance (24). Measurement of the blood glucose concentration on blood drawn from the (central) venous catheter through which insulin and glucose are administered will not be allowed due to potential interference with the measurement. The sponsor will keep a separate record of the used blood gas analyzers and glucometers used throughout the study in each center.

In accordance with the recent feeding guidelines for critically ill patients, enteral nutrition will be started as soon as possible (25). When enteral nutrition is insufficient to meet the caloric requirements, supplemental parenteral nutrition will not be initiated before day 8 in ICU, in accordance with recent evidence (17, 18). Except from a small amount of parenteral glucose, no other macronutrients will be administered by the parenteral route before day 8. Only in the most severely malnourished patients (BMI below 17 kg/m^2) and in patients readmitted to the ICU, parenteral nutrition can be initiated earlier, as these patients were excluded from the EPaNIC trial on supplemental parenteral feeding (17). However, there is no convincing evidence that this group of patients would benefit from early supplemental parenteral nutrition. In case early parenteral nutrition would be planned upon ICU admission in these patients, the patient will not be included in the trial. In general, the amount of parenteral glucose given during the first week in ICU by maintenance solutions will not exceed the equivalent amount of 1 ml dextrose 5% per kg per hour, unless the patient develops spontaneous hypoglycemia (hypoglycemia while not on insulin treatment) or has high risk to do so (e.g. in acute liver failure), or when the patient has a need for high volumes of hypotonic fluids (e.g. severe hypernatremia due to fluid losses). Until the patient receives at least 80% of his/her caloric needs of enteral nutrition, micronutrients (trace elements, minerals and vitamins) will be administered parenterally to prevent refeeding syndrome, according to local standard practice.

6.3 Laboratory tests

Whole blood glucose concentrations will be measured on arterial blood using a blood gas analyzer on each ICU and will be registered for later calculation of glucose metrics. Simultaneously, pH, blood gases, electrolytes (including potassium) and lactate are measured. When an arterial line is not in place, glucose will be measured on capillary blood using a validated glucometer. The frequency of blood glucose measurement depends on the blood glucose variability and on the advice given by the LOGIC-insulin algorithm, but minimally 4 to 6 measurements per day are required.

For the purpose of this clinical study, the day starts at 7:00 am. Blood samples will be taken upon ICU admission and daily at 06:00h ± 2 hours until discharge from ICU or death. Analyses on these samples will include, among others, routine clinical chemistry, hematology (Hgb, WBC, TC), markers of inflammation (CRP), liver function (ALT, AST, ALP, GGT, bilirubin total/direct), according to local practice. Other laboratory tests on these samples are performed on selected days, including several hormones, coagulation and fibrinolysis tests, cardiac biomarkers, triglycerides and creatine kinase, according to local practice. Upon ICU admission, hemoglobin A1c will be measured according to local practice. Urine samples will be taken daily at 06:00h ± 2 hours until discharge from ICU or death, according to local practice. Analysis of these urine samples includes urea and creatinine. According to clinical guidelines, blood cultures will be taken whenever central body temperature rises >38.5°C acutely or when other clinical signs of sepsis are present. These routine laboratory diagnostics make part of standard of care and will be performed by the central laboratory of the hospital.

In selected centers, upon ICU admission and daily at 06:00h ± 2 hours until discharge from ICU or death, extra samples will be taken and stored for determination of biochemical, metabolic, immunological, endocrine, inflammatory and (epi)genetic markers. This sampling will consist of 1 coagulated sample to spin for serum (yellow SST II tube [BD Vacutainer 367955], containing 5 ml of blood) and 1 EDTA sample to spin for plasma and cellular fraction (purple K2E tube [BD Vacutainer 368861], containing 4 ml of blood). The on-admission blood sampling will also include an additional coagulated sample to store. In selected centers, daily at 06:00h ± 2 hours until discharge from ICU or death, a 24-hour urine sample (or, at the first day, from ICU admission till the time of sampling) will be stored for determination of biochemical, metabolic, immunological, endocrine, inflammatory and (epi)genetic markers. A subset of the samples will be immediately stored on ice and processed in a cold chain for future endocrine and metabolic measurements. Processed serum and plasma as well as the spun-down blood cells and the sampled urine will be frozen at -20°C and stored at -80°C until further analysis. In selected centers and depending on additional funding, fresh pericardial and pleural fluid will be collected from patients admitted after cardiac surgery. These samples will be collected upon ICU admission and daily at 6:00h ± 2 hours until removal of pericardial and pleural drains or death. The macroscopic aspect of drainage fluid will be documented. Sampling

will consist of one Lithium-Heparin sample to spin for the acellular fraction (light green gel LiHeparine tube [BD vacutainer 367886], containing 4.5 mL of pericardial/pleural fluid) and one EDTA sample to spin for both cellular and acellular fractions (purple K2E tube [BD Vacutainer 368861], containing 4 ml of pericardial/pleural fluid). These samples will be immediately stored on ice and processed in a cold chain for future determination of metabolic, endocrine, inflammatory, biochemical, immunological, oxidative stress, cardiac and (epi)genetic markers – depending on funding. Processed samples will be frozen at -20°C and stored at -80°C until further analysis. This sampling strategy will not interfere with routine clinical practice. Patients are not exposed to additional invasive procedures, since insertion of a pericardial and pleural drain is standard practice after cardiac surgery. Pericardial and pleural drains will be removed according to standard clinical practice.

6.4 Other investigations

In selected centers, the presence of ICU-acquired weakness will be assessed clinically (see 1. Clinical assessment). In these centers, depending on funding, the clinical assessment will be supplemented by electrophysiological tests, ultrasound, functional tests and muscle biopsy at day 7±1, day 14±1 and day 21±1 in patients still in ICU at that time (see 2. till 5. Below). Additional informed consent will be asked for performing additional electrophysiological tests and muscle biopsies. The patient or his/her legal representative will have the possibility to only consent to a subset of these additional tests. Based on the results from previous studies and a lower anticipated effect size of blood glucose control in patients not receiving early parenteral nutrition, we expect a 12% difference in the electrophysiological incidence of critical illness polyneuropathy/myopathy (26, 27). With an α- and β-error of 5, respectively 20%, minimum 424 patients need to be included. The electrophysiological findings will be correlated with markers of muscle mass and tissue changes.

1. Clinical assessment
   In patients awake at day 7±1, day 14±1, day 21±1, day 28±1, and thereafter at ICU discharge, muscle function will be assessed with the Medical Research Council (MRC) sum score and a dynamometer will be used to measure handgrip strength. In selected centers, also clinical assessments performed as part of standard care will be recorded. The MRC sum score bilaterally evaluates strength in three upper (upper arm abductors, elbow flexors and wrist extensors) and three lower limb muscle groups (hip flexors, knee extensors and foot dorsal flexors) attributing a score between 0 and 5 (0: no contraction; 1: visible/palpable contraction without limb movement; 2: limb movement only with gravity eliminated; 3: limb movement is possible against gravity but not against resistance; 4: subnormal contraction against resistance; 5: normal muscle strength) to each of them, rendering a maximal total score of 60 (29). A cut-off of <48 has been shown to identify ‘significant weakness’ (30-32).

Handgrip strength will be measured provided that a minimum value of 3 is scored for both wrist extension and forearm flexion (28). Maximal inspiratory pressures will be recorded if monitored as part of standard care.

2. Electrophysiological tests

   Electrophysiological tests will be performed at day 7±1, day 14±1, and day 21±1in ICU. *Repetitive nerve stimulation* of the abductor digiti minimi muscle of the hand is performed at 3 Hz to evaluate the neuromuscular junction. *Nerve conduction studies* to evaluate the presence of peripheral neuropathy or myopathy include measurement of compound muscle action potential (CMAP) of the anterior tibial and abductor digiti minimi muscle and of sensory nerve action potential (SNAP) of the little finger under supramaximal stimulation with a hand-held stimulation probe. Presence of edema is scored. *Needle electromyography* (EMG) is performed in the resting condition in the tibialis anterior, rectus femoris, extensor digitorum communis and deltoideus pars media muscles to evaluate the presence of abundant spontaneous electrical activity (SEA), defined as the presence of sustained fibrillation potentials and/or positive sharp waves. *Direct muscle stimulation* of the tibialis anterior muscle is performed as previously described to differentiate between myopathy and neuropathy (33). The stimulation electrode is placed near the lower third of the tibialis anterior muscle. The recording electrode is positioned 30mm proximal to the stimulation electrode, guided by the twitch and depth at which maximal amplitude is obtained. Both stimulating and recording electrodes are repositioned until maximal response is obtained. Compound muscle action potential amplitudes generated after direct muscle stimulation (dmCMAP) are measured peak to peak. With the same recording electrode in place, the deep peroneal nerve is stimulated at the head of the fibula and the resulting motor response is recorded (nerve-stimulated CMAP, neCMAP). The ratio of neCMAP/dmCMAP will be calculated. These tests will be performed on the contralateral side of the eventual biopsy (see below). Also data obtained by electrophysiological tests performed as part of standard care will be recorded in selected centers.

3. Ultrasound

   In selected centers, ultrasound of the quadriceps muscle and the diaphragm will be performed at day 7±1, day14±1, and day 21±1 in patients still in the ICU at these time points. For ultrasound of the quadriceps muscle, a linear array commercial real-time ultrasound scanner will be used with a 12-MHz transducer. Ultrasonographic images will be collected from a transversal scan of the thigh with the patient in the supine position, with the knee extended and the muscle relaxed. The transducer will be placed perpendicular to the long axis of the thigh on three-fifths of the distance from the anterior superior iliac spine to the superior patellar border. This position will be defined beforehand with a surgical skin marker after measuring the exact position with a tape measure. An excess of contact gel will be applied to minimize image distortion. By obtaining maximal reflection of the bone, optimal transducer orientation is achieved and oblique scanning is minimized. All images will be

analyzed directly on the ultrasound scanner. The probe will be angled to obtain the highest density in the cortical bone of the femur. Then the pressure on the probe will be gradually released up to the point where the view on the outer margins is lost. At this point of minimal pressure, the muscle thickness in the center of the view will be measured. Muscle thickness will be defined as the distance between the superior border of the muscle and the cortex of the femur. Each limb will be measured. If feasible, also cross-sectional area of the quadriceps femoris, ultrasound intensity and pennation angle will be quantified.

For ultrasound of the diaphragm, the patient is positioned head-end of the bed 30° up. A 4-12 MHz linear array transducer is positioned at the zone of apposition between the anterior and mid-axillary lines at the level of the 9th or 10th intercostal space and angled perpendicular to the chest. The diaphragm is identified a three-layered structure just superficial to the liver, as a relative non-echogenic muscular layer, bounded by the echogenic membranes of the diaphragm pleura and peritoneum. After visualisation, the probe position is marked. Patients are assessed while receiving ventilator settings as set by the treating physician in case of assisted or controlled mechanical ventilation. For patients breathing spontaneously, the PS level is set to obtain TV 6-8ml/kg IBW, ensuring comfortable breathing. Measurements of end-expiratory thickness (Tdi,ee) and the following end-inspiratory thickness (Tdi,ei) are performed during 3 respiratory cycles. Diaphragm thickness is measured as the distance between the diaphragmatic pleura and the peritoneum using the M mode. Thickening fraction is calculated as TFdi = (Tdi,ei-Tdi, ee/Td,ee) for each of the three respiratory cycles, average value of these three calculations will be used for analyses. After the procedure, the mark is covered with a transparent cover that remains in place until the next measurement. In selected centers, ultrasound of the diaphragm will also be performed at baseline (day 0 or 1). Also data obtained by ultrasound of the diaphragm as performed as part of standard care will be recorded.

4. Functional tests

   Patients will be scored on the ICU mobility scale at day 7±1; day 14±1, and day 21±1 in ICU. In these patients, the ICU mobility scale will also be assessed at ICU discharge. This score represents a functional evaluation of the patient and varies from 0 to 10. Whereas a score of 0 represents a patient who is unable to move (lying in bed), score 10 represents a fully mobile patient (defined as walking at least 5 meters independently without a gait aid or assistance from another person) (34).

5. Muscle biopsy

   To correlate the clinical and electrophysiological findings to cellular changes, a small muscle and fat biopsy will be harvested at day 7±1, day 14±1, and day 21±1 in ICU. This will enable the study of the underlying cellular and molecular mechanisms leading to ICU-acquired muscle weakness and potentially other ICU complications. A quadriceps (vastus lateralis) needle biopsy will be taken under local anesthesia. Biopsies will not be obtained in patients with increased bleeding risk (platelet count <50000/µl, PT<40%, receiving anticoagulant

treatment or other bleeding diathesis). The skin is disinfected and sterile dressings are applied. Skin, subcutaneous tissues and muscle are infiltrated with lidocaine 2% solution via a 21 gauche injection needle. After 5 minutes, a 5 mm skin incision is made with a # 23 surgical blade (Swann-Morton). Subsequently, the needle biopsy will be taken in a sterile manner with a 5 mm Bergstrom needle. The subcutaneous adipose tissue of the quadriceps muscle biopsy will be stored separately. After manual hemostatic compression, the skin incision is closed with Steristrips™ (Nexcare™) skin closure adhesive strips and covered with a sterile transparent wound dressing. A compressive bandage is applied for 8 hours. An information letter with instructions for aftercare will be given to the nurse. In the muscle and subcutaneous adipose tissue, we will map crucial mediators of the different (epi)genetic, inflammatory, endocrine and metabolic pathways (depending on additional funding).

Depending on additional funding, additional investigations will be performed on the stored blood, urine and drainage fluid samples to unravel the metabolic, endocrine, inflammatory, immunological and (epi)genetic pathways involved in mediating organ dysfunction.

In selected centers, survival status will be investigated up to 4 years post randomization by consulting the National Registry (Rijksregister) or by other means for foreigners (checking the medical files). Depending on additional funding and in selected centers, subgroups of patients will be reinvited for a 1-2 yearly follow-up consultation up to 4 years post randomization. At that time point, recovery of organ function, functional status and muscle strength will be investigated by clinical history taking and clinical assessment, eventually supplemented by laboratory tests, questionnaires, functional testing and/or muscle biopsies. The protocol and informed consent for any follow-up investigation that is not routinely performed will be filed in an amendment of the current protocol.

7. Assessment of efficacy

The primary and secondary endpoints of the study are described in section 3 (study design).

8. Assessment of safety

8.1 Notification of adverse events and serious adverse events

Critical illness is a condition with adverse outcomes that are expected to occur. In this study adverse clinical outcomes are listed primary, secondary or safety endpoints. Hence, none of these study outcome endpoints will be considered as serious adverse events or suspected unexpected serious adverse reactions and thus do not need to be reported to the competent authorities. Any other and unexpected adverse reaction will be reported to the study sponsor.

For safety reasons, an interim analysis of hospital mortality will be performed after inclusion of 50% of the study population, with stopping boundaries predefined by the DMC to allow early study termination if the intervention group would appear clearly inferior.

The main adverse event of the intervention is hypoglycemia, which warrants prompt treatment. However, ICU physicians and nurses are familiar with the effects of insulin administration and follow-up of blood glucose concentrations. Hence, reporting hypoglycemic episodes to the competent authorities is not necessary. However, severe hypoglycemia (<40 mg/dl) that is resistant to intravenous glucose administration will be considered as a serious adverse event. This adverse event will be reported to the sponsor, after first knowledge. The immediate report will be followed by detailed, written reports. The sponsor will keep detailed records of all adverse events which are reported to him. These records will be submitted to the competent authorities when requested. In the intervention group, tight glucose control is timely stopped before ICU discharge, so that hypoglycemia provoked by the intervention is not expected to occur after ICU discharge. Therefore, hypoglycemia or other new medical events that would occur after ICU discharge and that would not be listed in the outcome measures will not be considered as adverse events, since these events cannot be attributed to short-acting insulin.

8.2 Treatment stopping rules

As mentioned above, the study intervention (tight blood glucose control) will be discontinued:
- when the patient resumes oral carbohydrate intake (meals, energy-rich drinks)
- when the central venous catheter is removed and not replaced
- upon ICU discharge

As long as the patient fulfills one of these stopping criteria, a liberal blood glucose control strategy will be maintained. When these stopping rules would cease in a patient, the study intervention will be restarted. This may occur when the patient would get sicker again while still in ICU (with placement of new arterial and venous line and interruption of oral feeding), or when the patient would be readmitted to the ICU within 48 hours after ICU discharge.

8.3 Data monitoring committee (DMC)

As mentioned below, a first interim analysis will take place after inclusion of 25% of the study population, to ensure adequate statistical power and adjust sample size if needed.

For safety reasons, an interim analysis of the safety endpoint (hospital mortality) will be performed after the first 4615 patients (50% of the study population), with stopping boundaries

predefined by the DMC to allow early study termination if the intervention would appear clearly inferior.

The independent DMC will also assess protocol compliance. To that purpose, the inclusion/exclusion rate and reasons for exclusion, the time profiles of blood glucose during the first 2 weeks in ICU, the incidence of hypoglycemia in ICU and the amount of enteral/parenteral feeding during the first week in ICU will be reviewed.

9. Statistics

9.1 Sample size

The sample size is calculated in order to detect, with at least 80% power (two-tailed) and 95% certainty, a reduction in ICU dependency by one day. With a baseline mean ICU stay of 9 days in ICU and a standard deviation of 15, 2782 patients in each group are needed to detect a reduction by one day (total 5564). For safety reasons, we also want to exclude any clinically relevant harmful impact on hospital mortality (safety endpoint). To detect an adversely increased hospital mortality from 8.5% to 10% with 80% power and 95% certainty, 4612 patients per group need to be included (total 9224). Hence, we plan to include 9230 patients.

The baseline mean ICU stay and its standard deviation are based on our previous multicenter RCT (17). As the baseline ICU stay may have changed over time, may differ per center and as it is difficult to predict the relative contribution of each center, we plan an interim analysis after inclusion of 25% of the study population (n=2308). At that time point, the independent data monitoring committee will receive an export of the necessary data (ICU length of stay and ICU mortality) in the control group (liberal blood glucose control) and will calculate a new sample size.

The primary endpoint (ICU dependency) will be analyzed in an unadjusted manner. The crude number of days with need for vital organ support will be compared by Student’s t test or non-parametric testing, as appropriate. The time to live discharge from ICU will be documented by Kaplan-Meier plots with use of log-rank/Wilcoxon testing; the time-to-event effect size will be estimated with the use of Cox proportional hazard analysis provided the proportional hazard assumption is valid for the dataset. The ICU mortality will be compared by chi-square test.

We anticipate that 2 years will be needed to take this study to completion (from first inclusion until last inclusion), provided informed consent will be at least 80%.

9.2 Randomization

The randomization procedure is described under heading 5.4. Patients will be stratified according to primary diagnostic category on ICU admission. Patients and family members will be blinded to treatment assignment. Due to the nature of the study, blinding physicians and nurses is not possible. Outcome assessors will be blinded, however.

9.3 Analysis

A Consolidated Standards of Reporting Trials (CONSORT) diagram will be reported.

For the primary and most secondary endpoints taking place during ICU stay, all data will be available. Of the secondary endpoints, some laboratory measurements may be missing. Missing data for one time point may be interpolated, by calculating the mean of the two neighbor measurements. In other cases, missing data will not be imputed.
All analyses will be done on intention to treat basis. In case of request for discontinuation of the study intervention by the patient or legal representative, this will be respected, but all data that are already collected will be analyzed.

The data file will be finalized and locked 90 days after inclusion of the last patient (except for the post ICU follow-up outcome parameters, as specified above). Discrete variables will be summarized by frequencies and percentages. Continuous variables will be summarized by use of either mean and standard deviation (SD) or median and interquartile range as appropriate. Results will be analyzed with the use of Chi-square testing, Student's t test or non-parametric testing (Wilcoxon rank-sum test, Van der Waerden test or Median test), as appropriate. Time-to-event effects will be documented by Kaplan-Meier plots with use of log-rank/Wilcoxon testing; the time-to-event effect size will be estimated with the use of Cox proportional hazard analysis provided the proportional hazard assumption is valid for the dataset. All time-to-event analyses will also be performed on data censored at 90 days. As death is a competing risk for duration of care outcomes, non-survivors will be censored beyond the longest duration of such care required for survivors.

All outcomes will be analyzed in an uncorrected manner as well as adjusted for baseline risk factors (including type and severity of illness, age, gender, body mass index, history of diabetes or not, center). For these analyses, P-values will be considered significant when at or below 0.05 without correction for multiple testing. To assess whether any eventual impact of the intervention on the primary and safety endpoints is affected by the baseline risk factor subgroup, interaction P-values will be calculated (logistic regression or Cox proportional hazard analysis as appropriate) with a threshold for significance of interaction set at a P-value of <0.1. These a

priori defined subgroups are: patients after cardiac surgery as compared with all other patients; patients admitted after elective surgery as compared with all other patients; patients admitted after surgery as compared with all other patients; patients with and without sepsis upon admission; patients with or without a known history of diabetes and/or an elevated HbA1c upon admission; patients admitted for a neurological/neurosurgical reason as compared with all other patients; patients admitted with brain injury as compared with all other patients; patients with high severity of illness score upon admission (APACHE-II ≥20) versus all other patients. Likewise, to study whether the intervention has a different effect in short-stay versus long-stay patients (ICU stay >3 days), an interaction P-value will be calculated.

Prior to study termination and analysis of the results on short-term outcome, a full statistical analysis plan will be published.

10. Quality assurance

To aid in achieving tight blood glucose control within the healthy fasting ranges and to reduce the risk of hypoglycemia and of blood glucose variability, blood glucose control will be guided by the LOGIC-insulin software. We previously demonstrated efficacy and safety of the LOGIC-insulin algorithm in a multicenter trial (20). As mentioned above, the software also advices on when to perform a next blood glucose measurement. The LOGIC-insulin software serves an advisory role. Eventually, the bedside nurse and physician have the possibility to overrule the given advice. However, our previous studies revealed a very high compliance rate.
As the difference in feeding regimen may be an important factor explaining the divergent outcomes of previous RCTs, the feeding intake needs to be standardized. In recent years, several studies have shown clinical advantage of a nutritional regimen that withholds parenteral nutrition in the acute phase of critical illness (first week in ICU).

11. Direct access to source data and documents

The sponsor will provide direct access to the case record form (CRF), the source data and the study master file for monitoring, independent Ethics Committee review and regulatory inspection.

12. Ethics and regulatory approvals

The trial will be conducted in compliance with the principles of the Declaration of Helsinki (2013), the principles of GCP and in accordance with all applicable regulatory requirements. This protocol and related documents will be submitted for review to the (local) Ethics Committee and to the Federal Agency for Medicine and Health Products for Clinical Trial

Authorization. Any subsequent protocol amendment will be submitted to the (local) Ethics Committee and Regulatory Authorities for approval. Required progress reports and a copy of the final study report will be provided to the (local) Ethics Committee and the Federal Agency for Medicine and Health Products.

The Study can and will be conducted only on the basis of informed consent by the subjects or their legal representatives to participate in the Study. The Participating Site will perform maximal efforts to obtain a signed informed consent form (ICF) for all patients within 72 hours after their enrollment and participation in the Study in compliance with all applicable laws, regulations and the approval of the (local) Ethics Committee. If necessary, the legal representative will be contacted by phone to obtain informed consent, which will be followed by a request to resend a written informed consent. The Participating Site will retain such ICFs in accordance with the requirements of all applicable regulatory agencies and laws.

The Investigator and the Participating Site shall treat all information and data relating to the Study disclosed to Participating Site and/or Investigator in this Study as confidential and shall not disclose such information to any third parties or use such information for any purpose other than the performance of the Study. The collection, processing and disclosure of personal data, such as patient health and medical information is subject to compliance with applicable personal data protection and the processing of personal data (Directive 95/46/EC and Belgian law of December 8, 1992 on the Protection of the Privacy in relation to the Processing of Personal Data)

Electronic data collection will be used. Data will be collected in a CRF, unambiguously linked to the source file. For reasons of data integrity and internal control during data input, the patient name is stored in a separate table linked to the eCRF. However, these data will only be accessible for the authorized local research staff and the principal database manager on a login/password base. When the database is finalized, the identity data will be blinded from the eCRF and only accessible by the study monitor or his substitute.

The sponsor will provide direct access to the CRF, the source data and the study master file for monitoring, independent Ethics Committee review and regulatory inspection. The sponsor will establish a data monitoring committee. The sponsor appointed a monitor (Lies Langouche). The monitor will verify that the trial is performed in accordance to the protocol as described in the European Medicine Agency's 'Note for guidance on good clinical practice CPMP/ICH/135/95' as well as the Declaration of Helsinki.

13. Data handling

Screening procedures will take place as soon as possible after admission to the ICU (within 2 hours). A screening number will be assigned to the patient in ascending order. The following data will be recorded in the CRF after informed consent is obtained:

- Patient number

- Patient name: for reasons of data integrity and internal control during data input, the patient name is stored in a separate table linked to the eCRF. However, these data will only be accessible for the authorized local research staff and the principal database manager on a login/password base. When the database is finalized, the identity data will be blinded by the database manager.
- Hospital ID number
- Randomization group
- Randomization number
- Unit & bed number
- On admission diagnosis group
- Admission for brain injury (Y/N) and type (traumatic brain injury, subarachnoid hemorrhage, intracranial bleeding, ischemic stroke, out-of-hospital cardiac arrest)
- Data of birth and age
- Gender
- Admission date and time
- Height, weight, body mass index (BMI)
- Emergency admission (Y/N)
- APACHE-II score after 24 hours
- Diagnosis (reason for ICU admission)
- Diagnostic category
- Infection on admission (Y/N)
- History of diabetes (Y/N, type, R/diet, R/OAD, R/insulin), HbA1c, with or without end-organ damage (retinopathy, neuropathy, nephropathy, brittle diabetes)
- History of dyslipidemia (Y/N, R/diet, R/lipid-lowering drugs) (selected centers)
- History of COPD (Y/N), Gold classification; other chronic pulmonary disease (Y/N)
- History of myocardial infarction (not ECG changes only) (Y/N); History of heart failure (Y/N); NYHA classification
- Last ejection fraction (selected centers)
- History of hypertension (Y/N, systolic blood pressure consistently > 140 mmHg and/or diastolic blood pressure consistently > 90 mmHg or treated hypertension on medication)
- History of atrial fibrillation (Y/N) (selected centers)
- CHA2DS2-VASc score (0-9) (selected centers)
- Implantable Cardiac Rhythm Device prior admission (Y/N) (selected centers)
    - Pacemaker (Y/N)
    - Implantable Cardioverter Defibrillator (ICD) (Y/N)
    - Cardiac Resynchronization Therapy Pacemaker (CRT-P) (Y/N)
    - Cardiac Resynchronization Therapy Defibrillator (CRT-D) (Y/N)
- Peripheral vascular disease (includes aortic aneurysm ≥6cm) (Y/N)
- Liver disease

    - portal hypertension
    - hepatic cirrhosis
- Neurological disease
    - Dementia
    - History of stroke (Y/N) (cerebrovascular accident (CVA) and/or transient ischemic attack (TIA)) (selected centers)
    - Cerebrovascular disease with mild or no disability
    - Hemiplegia
    - Paraplegia
- History of active malignancy (last 5 years):
    - Solid tumor without metastasis (Y/N)
    - Metastatic cancer (Y/N)
    - Hematological malignancy (Y/N)
- Chronic neuroleptics (Y/N)
- AIDS (not just HIV-positive) (Y/N)
- Peptic ulcer disease (Y/N)
- Connective tissue disease (Y/N)
- Immune suppressed status
    - Neutropenia (<1000 neutrophils/mm3)
    - corticosteroid therapy (prednisolone or equivalent >0.5 mg/kg/day for >3 months)
    - systemic corticosteroid therapy <3 months (lower dose)
    - chemotherapy within one year
    - radiotherapy within one year
    - bone marrow recipient
    - solid organ transplant recipient
    - immunosuppressive drug for auto-immune diseases
    - congenital immunodeficiency
- life style risk factors
    - tobacco use (>20 pack years)
    - alcohol abuse (>1L of wine /day or equivalent = 10g alcohol day)
    - IV drug abuse
- Insulin drip at admission (Y/N)
- Lowest plasma creatinine in 3 months period before ICU admission
- Last plasma creatinine before randomization (maximum 24 hours before ICU admission)
- Renal replacement therapy pre-admission (Y/N), chronic renal replacement therapy (Y/N)
- Blood glucose concentration on admission
- Blood lactate on admission
- Investigation site
- Investigation ID
- Check for inclusion criteria

- Adult patient (≥18 years of age)

- Check for exclusion criteria
  - Do not resuscitate (DNR) order or moribund (expected to die within 12 hours) at ICU admission
  - Patient able to receive oral feeding
  - No arterial line or central venous line needed
  - Previous inclusion in the trial
  - Enrolled in another outcome randomized controlled trial (RCT)
  - Transfer from a non-participating ICU with a pre-admission ICU stay >7 days
  - Planned to receive early parenteral nutrition (i.e. during the first week in ICU)
  - Diabetic ketoacidotic or hyperosmolar coma on ICU admission
  - Inborn metabolic diseases
  - Insulinoma
  - Known to be pregnant or lactating
- Informed consent (patient or relative)

A separate log file will be kept for the patients who were not included in the study. These data will not be used for analysis, but are required to construct an adequate patients' flow diagram (CONSORT diagram). Only the following data will be recorded:

- Hospital ID number
- Age, gender
- Admission date
- Diagnostic group
- Brain injury Y/N and subtype
- Reason for non-inclusion

In the admission file of the CRF, the data on the end of the study will also be included:

- End of study date, time and reason (stopping criteria)
- ICU discharge date (defined as ready to be discharged), ICU length of stay
- Hospital discharge date, hospital length of stay
- Readmission within 48 hours after discharge (Y/N)
- New infection during ICU stay (Y/N), type (bacteremia, airway, urinary tract, wound, other)
- Atrial fibrillation during ICU stay (Y/N) (recorded in selected centers)
- Major Adverse Cardiovascular Event (MACE) during ICU stay (Y/N) (not present on admission / not admission diagnosis) (recorded in selected centers):
  - Non-fatal myocardial infarction (Y/N)
  - Non-fatal stroke (Y/N)
  - Cardiovascular death (Y/N)

    - Low cardiac output (Y/N), i.e. cardiac index <2.5 l/min/m², need for inotropic support, need for mechanical circulatory support
- Survival outcome:
    - Mortality during hospitalization, Y/N
    - Mortality during ICU stay, Y/N
    - Mortality at day 90 (Y/N), date of death
    - Circumstances of death: therapy withdrawal, refractory shock, brain death
- File completed (Y/N)
- Authorization for completion

Simultaneously, all data from the patient data management system, which are used in routine clinical care, will be kept for the study patients. Additionally, the data that are highly relevant for the study will be included in the CRF on a daily basis:
- Blood glucose values. Only date and time stamped arterial and capillary blood glucose values will be included.
- Hypoglycemia report (will be recorded per hypoglycemic event <40 mg/dl)
    - Minimum blood glucose concentration
    - Maximum blood glucose concentration within 4 hours after correction of hypoglycemia
    - Date and time of hypoglycemia
    - Cause of hypoglycemia
    - Action undertaken
    - Insulin drip decrease/stop (Y/N/no insulin)
    - Bolus glucose (Y/N)
    - Blood glucose concentration >40 mg/dl within 1 hour (Y/N/no value)
    - Immediate consequences (Y/N/unclear)
        - Perspiration (Y/N)
        - Epilepsy within 8 hours (Y/N/Unclear)
        - Severe arrhythmia or shock (Y/N/pre-existing)
        - Death within 24 hours (Y/N) and duration (in hours) from hypoglycemia to death
        - Loss of consciousness (Y/N/sedation)
- All medication given systemically (cumulative daily dose though each route)
- Nutrition
    - Parenteral nutrition (kilocalories, amino acids, lipids and carbohydrates cumulatively administered during 1 day)
    - Enteral nutrition (kilocalories, amino acids, lipids and carbohydrates cumulatively administered during 1 day)
- Mechanical ventilation (Y/N)

- Tracheostomy (Y/N)
- VV ECMO (Y/N)
- Mechanical hemodynamic support (Y/N), type (VA ECMO, VAD (including Heartmate, HeartWare, Impella, Excor), IABP)
- Markers of renal function
    - Plasma urea and creatinine
    - Creatinine clearance
    - Urinary output (24 hours, or for the first day from ICU admission until the time of sampling)
- Markers of hepatic (dys)function/damage
    - Transminases
    - γ-glutamyltransferase
    - Alkaline phosphatase
    - Bilirubin (total/direct)
- Circulating cardiac biomarkers (in selected centers)
    - Troponin, CK-MB, CK
    - NTproBNP
- C-reactive protein
- Blood lactate (maximum value)
- Blood counts
    - Red blood cells count and hemoglobin level
    - Leukocyte count
    - Thrombocyte count
- SOFA score and the different components (respiration, coagulation, liver, cardiovascular, central nervous system, renal)
- Maximum temperature and minimum temperature
- Heart rate >90 per minute (Y/N)
- Minimum and maximum heart rate, need for cardiac pacing (Y/N), need for cardioversion (Y/N), presence of atrial fibrillation (Y/N); cardiac index, including minimum, mean/median and maximum value in patients with pulmonary artery catheter or other transpulmonary thermodilution device (PiCCO,…); mixed venous oxygen saturation (SvO_2), including minimum, mean/median and maximum value in patients with pulmonary artery catheter (in selected centers)
- Respiratory rate >20 per minute or arterial CO_2 pressure <32mm Hg (Y/N)
- ICSDC score (worst observation during the day) in selected centers
- MRC-sum score and handgrip strength in selected centers (at day 7±1, day 14±1, day 21±1, day 28±1 and last day in ICU). The 'last day in ICU' measurement will only be recorded in patients having undergone a previous assessment.

- In selected centers: ICU mobility scale score at day 7±1, day 14±1, day 21±1, day 28±1 and last day in ICU. The ‘last day in ICU’ assessment will only be recorded in patients having undergone a previous assessment.
- In selected centers: electrophysiological assessment at day 14±1, day 21±1 and day 28±1 in ICU
- In selected centers: ultrasonographic quadriceps muscle thickness left and right (at day 14±1, day 21±1 and day 28±1 in ICU)

For the post-ICU outcomes, a separate database will be created. Hence, the database with short-term clinical outcomes can be locked (after finalization and quality checks) 90 days after inclusion of the last patient.

Acute kidney injury (AKI) will be classified according to modified KDIGO criteria (21, 22). The baseline creatinine concentration is the lowest plasma creatinine in the three months preceding ICU admission. In case no baseline value is available, a baseline value will be imputed by backcalculation from the MDRD formula. If feasible, the hourly urinary output will be exported from the patient data management system.

As some patients may already have a significantly altered renal function upon ICU admission by pre-admission insults, we will also investigate the influence of the intervention on the evolution of the renal function after randomization. Therefore, we will record the last creatinine measurement before randomization, within a time window of maximum 24 hours before ICU admission. In case such measurement is not available, the first measurement in ICU will be considered to be the creatinine value upon randomization.

As described above, the repeated blood and urine measurements that are stored in the CRF are routine measurements during ICU stay (section 6).

All new infections and the type of infection (airway, bloodstream, urinary tract, wound and other) will be recorded in consensus by two experts in infectious diseases blinded to treatment assignment. All patients receiving antimicrobial agents will be identified by the data manager, who will provide an export of all patient numbers with all the information on antimicrobial agents given as well as the duration of such treatment. Then, the presence of infections will be identified by an infectious disease specialist who will be blinded for treatment allocation. All patients receiving antimicrobial agents for more than 48 hours will be selected. Patients receiving only antimicrobial prophylaxis will be excluded. Each patient who fulfills the criteria for infection, as well as the type of infection, will be identified as such based on thorough review of the medical record (35). Patients for whom antimicrobials where initiated prior to ICU admission or within the first 48 hours of admission while the criteria for infection were fulfilled,

will be labeled as ‘having an infection upon admission’. When antimicrobial agents were initiated after randomization and beyond the first 48 hours in the ICU, and were given for more than 48 hours while the criteria for infection were fulfilled, the patient will be labeled as ‘having a new infection’. Sepsis will be scored using the Bone criteria and by the Sepsis-3 criteria (provided this information is available in all centers) .

All medication given systemically to the patient during ICU stay will be registered. Every day, the amount of kilocalories, lipids, proteins, carbohydrates delivered by either parenteral or enteral nutrition will be entered into the CRF. Of the gastric residual volume discarded, half of the volume will be considered to be EN and half to be gastric secretions.

Data will be collected electronically in a coded electronic case record form (eCRF), unambiguously linked to the source file. Data will be manually or semi-automatically transferred and checked for accuracy into the eCRF by the clinical research assistants’ team on a daily basis. Routine laboratory results will be imported electronically. Extensive range and consistency checks will be performed by the study monitor. All original records, such as consent forms and relevant correspondence, will be archived at the participating centers, according to local regulations. Vital status at 90 days (and at later follow-up times) will be recorded for all patients, by the National Registry. When this information is not available, vital status will be checked through the hospital information system or the regional network of hospital physicians and general practitioners.

14. Data management

As mentioned above, an eCRF will be used. The eCRF is coded and unambiguously linked to the source file. For reasons of data integrity and internal control during data input, the patient name is stored in a separate table linked to the eCRF. However, these data will only be accessible for the authorized local research staff and the principal database manager on a login/password base. When the database is finalized, the identity data will be blinded and only accessible by the study monitor or his representative. The database will be finalized and locked 90 days after inclusion of the last patient, except for the longer term post-ICU outcomes. Patients still in the ICU or in the hospital at closing of the data file (90 days after last patient inclusion) will be censored at that time point. The database software is Filemaker Pro (FileMaker Inc, FileMaker International).
Investigators involved in the trial will not have direct access to the database. In addition, the study monitor will log the use of the database. After the trial, the study monitor will store all data in a secured file that is only accessible by the study monitor or his representative.

15. Translational research

In selected centers, as described above (section 6.2 and 6.3), blood, urine, pericardial and pleural fluid samples, as well as tissue samples will be harvested, frozen at -20°C and stored at -80°C until further analysis.

16. Publication policy

Publication policy will be addressed in separate agreements.

17. Insurance/indemnity

In accordance with the Belgian Law relating to experiments on human persons dated May 7, 2004, Sponsor shall assume, even without fault, the responsibility of any damages incurred by a Study Patient and linked directly or indirectly to the participation to the Study, and shall provide compensation therefore through its insurance.” A no fault insurance is covered by Amlin Corporate Insurance.

18. Financial aspects

Financial support is covered by grant from the Research Foundation – Flanders (TBM project T003617N). Jan Gunst receives a postdoctoral fellowship from the UZ Leuven Clinical Research Fund for this project.
Financial aspects between participating centers will be addressed in separate agreements.

19. Appendices

- Appendix 1: Summary of Product Characteristics
- Appendix 2: Informed consent and opting out forms (in Dutch)
- Appendix 3: Informed consent and opting out forms (in French)
- Appendix 3: Informed consent and opting out forms (in English)

20. References

1. Herridge MS, Tansey CM, Matte A, Tomlinson G, Diaz-Granados N, Cooper A, et al. Functional disability 5 years after acute respiratory distress syndrome. N Engl J Med. 2011;364(14):1293-304.

2. Vanderheyden S, Casaer MP, Kesteloot K, Simoens S, De Rijdt T, Peers G, et al. Early versus late parenteral nutrition in ICU patients: cost analysis of the EPaNIC trial. Crit Care. 2012;16(3).
3. Falciglia M, Freyberg RW, Almenoff PL, D'Alessio DA, Render ML. Hyperglycemia-related mortality in critically ill patients varies with admission diagnosis. Crit Care Med. 2009;37(12):3001-9.
4. Kosiborod M, Rathore SS, Inzucchi SE, Masoudi FA, Wang Y, Havranek EP, et al. Admission glucose and mortality in elderly patients hospitalized with acute myocardial infarction: implications for patients with and without recognized diabetes. Circulation. 2005;111(23):3078-86.
5. Van den Berghe G, Wouters P, Weekers F, Verwaest C, Bruyninckx F, Schetz M, et al. Intensive insulin therapy in critically ill patients. N Engl J Med. 2001;345(19):1359-67.
6. Ingels C, Debaveye Y, Milants I, Buelens E, Peeraer A, Devriendt Y, et al. Strict blood glucose control with insulin during intensive care after cardiac surgery: impact on 4-years survival, dependency on medical care, and quality-of-life. Eur Heart J. 2006;27(22):2716-24.
7. Van den Berghe G, Wouters PJ, Kesteloot K, Hilleman DE. Analysis of healthcare resource utilization with intensive insulin therapy in critically ill patients. Crit Care Med. 2006;34(3):612-6.
8. Van den Berghe G, Wilmer A, Hermans G, Meersseman W, Wouters PJ, Milants I, et al. Intensive insulin therapy in the medical ICU. N Engl J Med. 2006;354(5):449-61.
9. Vlasselaers D, Milants I, Desmet L, Wouters PJ, Vanhorebeek I, van den Heuvel I, et al. Intensive insulin therapy for patients in paediatric intensive care: a prospective, randomised controlled study. Lancet. 2009;373(9663):547-56.
10. Mesotten D, Gielen M, Sterken C, Claessens K, Hermans G, Vlasselaers D, et al. Neurocognitive development of children 4 years after critical illness and treatment with tight glucose control: a randomized controlled trial. JAMA. 2012;308(16):1641-50.
11. Finfer S, Blair D, Bellomo R, McArthur C, Mitchell I, Myburgh J, et al. Intensive versus conventional glucose control in critically ill Patients. N Engl J Med. 2009;360(13):1283-97.
12. Finfer S, Liu B, Chittock DR, Norton R, Myburgh JA, McArthur C, et al. Hypoglycemia and risk of death in critically ill patients. N Engl J Med. 2012;367(12):1108-18.
13. Association AD. 13. Diabetes care in the hospital. Diabetes Care. 2016;39 Suppl 1:S99-104.
14. Rhodes A, Evans LE, Alhazzani W, Levy MM, Antonelli M, Ferrer R, et al. Surviving Sepsis Campaign: International guidelines for management of sepsis and septic shock: 2016. Intensive Care Med. 2017.
15. Jacobi J, Bircher N, Krinsley J, Agus M, Braithwaite SS, Deutschman C, et al. Guidelines for the use of an insulin infusion for the management of hyperglycemia in critically ill patients. Crit Care Med. 2012;40(12):3251-76.

16. Niven DJ, Rubenfeld GD, Kramer AA, Stelfox HT. Effect of published scientific evidence on glycemic control in adult intensive care units. JAMA Intern Med. 2015;175(5):801-9.
17. Casaer MP, Mesotten D, Hermans G, Wouters PJ, Schetz M, Meyfroidt G, et al. Early versus late parenteral nutrition in critically ill adults. N Engl J Med. 2011;365(6):506-17.
18. Fivez T, Kerklaan D, Mesotten D, Verbruggen S, Wouters PJ, Vanhorebeek I, et al. Early versus late parenteral nutrition in critically ill children. N Engl J Med. 2016;374(12):1111-22.
19. Van Herpe T, Mesotten D, Wouters PJ, Herbots J, Voets E, Buyens J, et al. LOGIC-Insulin algorithm-guided versus nurse-directed blood glucose control during critical illness: the LOGIC-1 single-center, randomized, controlled clinical trial. Diabetes Care. 2013;36(2):188-94.
20. Mesotten D, Dubois J, Van Herpe T, van Hooijdonk RT, Wouters R, Coart D, et al. Software-guided versus nurse-directed blood glucose control in critically ill patients: the LOGIC-2 multicenter randomized controlled clinical trial. Crit Care. 2017;21(1):212.
21. Kidney Disease Improving Global Outcomes (KDIGO) Acute Kidney Injury Work Group. KDIGO clinical practice guideline for acute kidney injury. Kidney Int. 2012;Suppl 2:1-138.
22. Gunst J, Vanhorebeek I, Casaer MP, Hermans G, Wouters PJ, Dubois J, et al. Impact of early parenteral nutrition on metabolism and kidney injury. J Am Soc Nephrol. 2013;24(6):995-1005.
23. Gunst J, Van den Berghe G. Blood glucose control in the intensive care unit: benefits and risks. Semin Dial. 2010;23(2):157-62.
24. Claerhout H, De Prins M, Mesotten D, Van den Berghe G, Mathieu C, Van Eldere J, et al. Performance of strip-based glucose meters and cassette-based blood gas analyzer for monitoring glucose levels in a surgical intensive care setting. Clin Chem Lab Med. 2016;54(1):169-80.
25. Reintam Blaser A, Starkopf J, Alhazzani W, Berger MM, Casaer MP, Deane AM, et al. Early enteral nutrition in critically ill patients: ESICM clinical practice guidelines. Intensive Care Med. 2017;43(3):380-98.
26. Van den Berghe G, Wilmer A, Milants I, Wouters PJ, Bouckaert B, Bruyninckx F, et al. Intensive insulin therapy in mixed medical/surgical intensive care units - Benefit versus harm. Diabetes. 2006;55(11):3151-9.
27. Hermans G, Van Mechelen H, Bruyninckx F, Vanhullebusch T, Clerckx B, Meersseman P, et al. Predictive value for weakness and 1-year mortality of screening electrophysiology tests in the ICU. Intensive Care Med. 2015;41(12):2138-48.
28. Hermans G, Clerckx B, Vanhullebusch T, Segers J, Vanpee G, Robbeets C, et al. Interobserver agreement of medical research council sum-score and handgrip strength in the intensive care unit. Muscle Nerve. 2012;45(1):18-25.

29. Kleyweg RP, Vandermeche FGA, Schmitz PIM. Interobserver agreement in the assessment of muscle strength and functional abilities in Guillain-Barré syndrome. Muscle Nerve. 1991;14(11):1103-9.
30. De Jonghe B, Sharshar T, Lefaucheur JP, Authier FJ, Durand-Zaleski I, Boussarsar M, et al. Paresis acquired in the intensive care unit - A prospective multicenter study. JAMA. 2002;288(22):2859-67.
31. De Jonghe B, Bastuji-Garin S, Sharshar T, Outin H, Brochard L. Does ICU-acquired paresis lengthen weaning from mechanical ventilation? Intensive Care Med. 2004;30(6):1117-21.
32. Ali NA, O'Brien JM, Hoffmann SP, Phillips G, Garland A, Finley JCW, et al. Acquired weakness, handgrip strength, and mortality in critically ill patients. Am J Respir Crit Care Med. 2008;178(3):261-8.
33. Rich MM, Teener JW, Raps EC, Schotland DL, Bird SJ. Muscle is electrically inexcitable in acute quadriplegic myopathy. Neurology. 1996;46(3):731-6.
34. Hodgson C, Needham D, Haines K, Bailey M, Ward A, Harrold M, et al. Feasibility and inter-rater reliability of the ICU Mobility Scale. Heart Lung. 2014;43(1):19-24.
35. Horan TC, Andrus M, Dudeck MA. CDC/NHSN surveillance definition of health care-associated infection and criteria for specific types of infections in the acute care setting. Am J Infect Control. 2008;36(5):309-32.
36. Singer M, Deutschman CS, Seymour CW, Shankar-Hari M, Annane D, Bauer M, et al. The third international consensus definitions for sepsis and septic shock (Sepsis-3). JAMA. 2016;315(8):801-10.
